# Platelet-rich plasma as a potential antimicrobial agent against multidrug-resistant bacteria in diabetic foot infections

**DOI:** 10.1038/s41598-025-97418-0

**Published:** 2025-04-30

**Authors:** Engy Aboelsaad, Sameh Moustafa, Amira Amine, Akram Deghady, Laila El-Attar

**Affiliations:** 1 Department of Microbiology, High Institute of Public Health, Alexandria University, El-Horreya Road 165, Alexandria, 21561 Egypt; 2 Department of Vascular Surgery, Faculty of Medicine, Alexandria University, Chamblion Street, Alexandria, 21521 Egypt; 3 Department of Clinical and Chemical Pathology, Faculty of Medicine, Alexandria University, Chamblion Street, Alexandria, 21521 Egypt

**Keywords:** Antimicrobial agents, Platelet-rich plasma, Multidrug-resistance, Diabetic foot, Infectious diseases, Antimicrobials, Bacteriology

## Abstract

Diabetes mellitus is a global public health concern, with diabetic foot infections (DFIs) being common clinical complications among affected patients. Bacterial isolates resistant to commonly used antimicrobial drugs are becoming more prevalent in DFIs. Some research suggests that platelet-rich plasma (PRP) may inhibit bacterial growth, making it a promising biological therapy. Therefore, an in vitro experimental study was conducted on 53 multidrug-resistant (MDR) bacterial strains isolated from DFIs. The isolates were methicillin-resistant *Staphylococcus aureus* (MRSA), MDR *Klebsiella pneumoniae*, and MDR *Pseudomonas aeruginosa*. The antibacterial activity of PRP was assessed using Kirby-Bauer disk diffusion method, broth microdilution method, checkerboard synergy testing, and time-kill assay. The time-kill assay demonstrated that PRP’s antibacterial efficacy peaked during the second hour of incubation for MRSA and *Pseudomonas aeruginosa*, but peaked at the first hour for *Klebsiella pneumoniae*. However, the PPR’s efficiency against all isolates decreased after the peak point, with no antibacterial activity observed at the 24th h of incubation. Additionally, biofilm inhibition and eradication assays revealed that PRP has no effect on biofilm formation. As a result, PRP has the ability to inhibit bacterial growth, although this effect is transient and depends on the bacterial strain.

## Introduction

Diabetes mellitus (DM) constitutes a significant public health issue, ranking among the most prevalent and serious chronic diseases of the 21st century^1^. Complications of DM, which are frequently debilitating, life-threatening, and financially burdensome, are rising in both developed and developing nations^2^. The latest statistics from the 10th edition of the International Diabetes Federation (IDF) Diabetes Atlas indicates that around 536.6 million adults are presently affected by DM, with projections suggesting an increase to 783.2 million by 2045^3^.

Patients with DM face a 25% risk of developing foot ulcers, half of which are infected at diagnosis, leading to substantial morbidity, with 1 in 5 cases culminating in lower extremity amputation^4,5^. Diabetic foot infections (DFIs) can be caused by bacteria from the external environment as well as from the normal flora of the skin. Notably, multidrug-resistant (MDR) bacteria, such as *Pseudomonas aeruginosa* (*P. aeruginosa*), *Klebsiella pneumoniae* (*K. pneumoniae*), and methicillin-resistant *Staphylococcus aureus* (MRSA), are prevalent in DFIs. These MDR bacteria are virulent pathogens that resist most conventionally prescribed antibiotics. They are also considered to be among the clinically significant pathogens involved in biofilm formation associated with infections, which complicates ulcer management and increases healthcare costs^6,7^.

Platelet-rich plasma (PRP) is an autologous derivative of whole blood, with platelet concentrations higher than normal. It contains a complete set of clotting factors, cytokines, chemokines, and other plasma proteins^8–10^. It is also rich in growth factors (GFs), so it shows promise in the healing of various organs. Consequently, its application has significantly expanded in dermatology, reconstructive plastic surgery, orthopedics, spine surgery, sports medicine, oral and maxillofacial surgery, and ophthalmology^7,11^.

Current evidence suggests that PRP is a good antimicrobial agent that could aid in preventing infections and treating chronic wounds or bone infections^12–15^. The advantages of PRP include its affordability, ease of preparation, low likelihood of inducing antibiotic resistance, and autologous nature, ensuring safety^16^. Additionally, it has demonstrated efficacy against biofilm-producing microorganisms, which is essential for treating infections effectively^17,18^.

Despite the numerous advantages associated with PRP and the promising outcomes reported for its therapeutic potential, the clinical results vary widely and can even be contradictory. These conflicting findings arise from differences in the clinical protocols used, making it challenging to compare results and establish definite conclusions regarding their actual effectiveness. Additionally, the lack of standardization in PRP preparation procedures further highlights this issue. As a result, a wide array of products with various cell types, quantities, GFs, cytokine contents, and release times are available^19–21^.

Although PRP has been suggested as a treatment for clean diabetic foot ulcers (DFUs) and studies have revealed the antimicrobial activity of PRP on various microorganisms, such as *Streptococcus oralis* and *Candida albicans*^11,12,22,23^, limited research has examined resistant microorganisms that may endure prolonged periods and are challenging to manage in wound infections^17,24^. Consequently, further investigation is required to evaluate the in vitro antibacterial efficacy of PRP against MDR bacteria, facilitating prospective clinical studies and targeted PRP therapies in DFIs.

## Results

The present study was conducted over the period from April 2021 to May 2022 in the Microbiology Laboratory at at the High Institute of Public Health (HIPH) and the Clinical Pathology Laboratory at Alexandria Main University Hospital (AMUH) to determine the antimicrobial activity of PRP against 53 MDR bacteria isolated from infected diabetic foot ulcers.

Samples were collected from 78 DFIs of patients admitted to the Vascular Surgery and Diabetic Foot Unit at the Surgery Department in AMUH. All samples were subjected to the standard microbiological procedures for isolation and identification of bacterial agents. Bacterial isolates other than MRSA, MDR *K. pneumoniae*, and MDR *P. aeruginosa* were excluded from the PRP experimental study.

Among 21 MDR *K. pneumoniae* isolates, 11 (52.40%) were extended-spectrum beta-lactamase (ESBL) producers, and among 16 MDR *P. aeruginosa* isolates, 6 (37.50%) were carbapenemase producers (Table 1).

**Table 1:** Distribution of the 53 studied bacterial isolates, Alexandria, 2021–2022.

Studied bacterial isolates		No.	%
MRSA		16	30.19
MDR K.* pneumoniae* (*n* = 21) (39.62%)	ESBL-producers	11	20.75
	Non ESBL-producers	10	18.87
MDR P.* aeruginosa* (*n* = 16) (30.19%)	Carbapenemase-producers	6	11.32
	Non carbapenemase-producers	10	18.87
Total		53	100.00

For PRP preparation, venous blood samples were obtained from 16 adult volunteers. The volunteers were all females, and the mean age was 31.17 ± 2.79 years. The mean platelet count was 233.17 ± 79.81 × 10^9^/L in whole blood, and increased to 823.67 ± 282.05 × 10^9^/L in PRP, with an average increase of 3.53-fold in the platelet concentration after processing. The mean white blood cells (WBCs) count of whole blood was 6.48 ± 2.23 × 10^9^/L, and was markedly reduced to 2.31 ± 5.03 × 10^9^/L in PRP.

### Determination of the antibacterial activity of PRP

#### Kirby–Bauer disk diffusion method

**Fig. 1:**
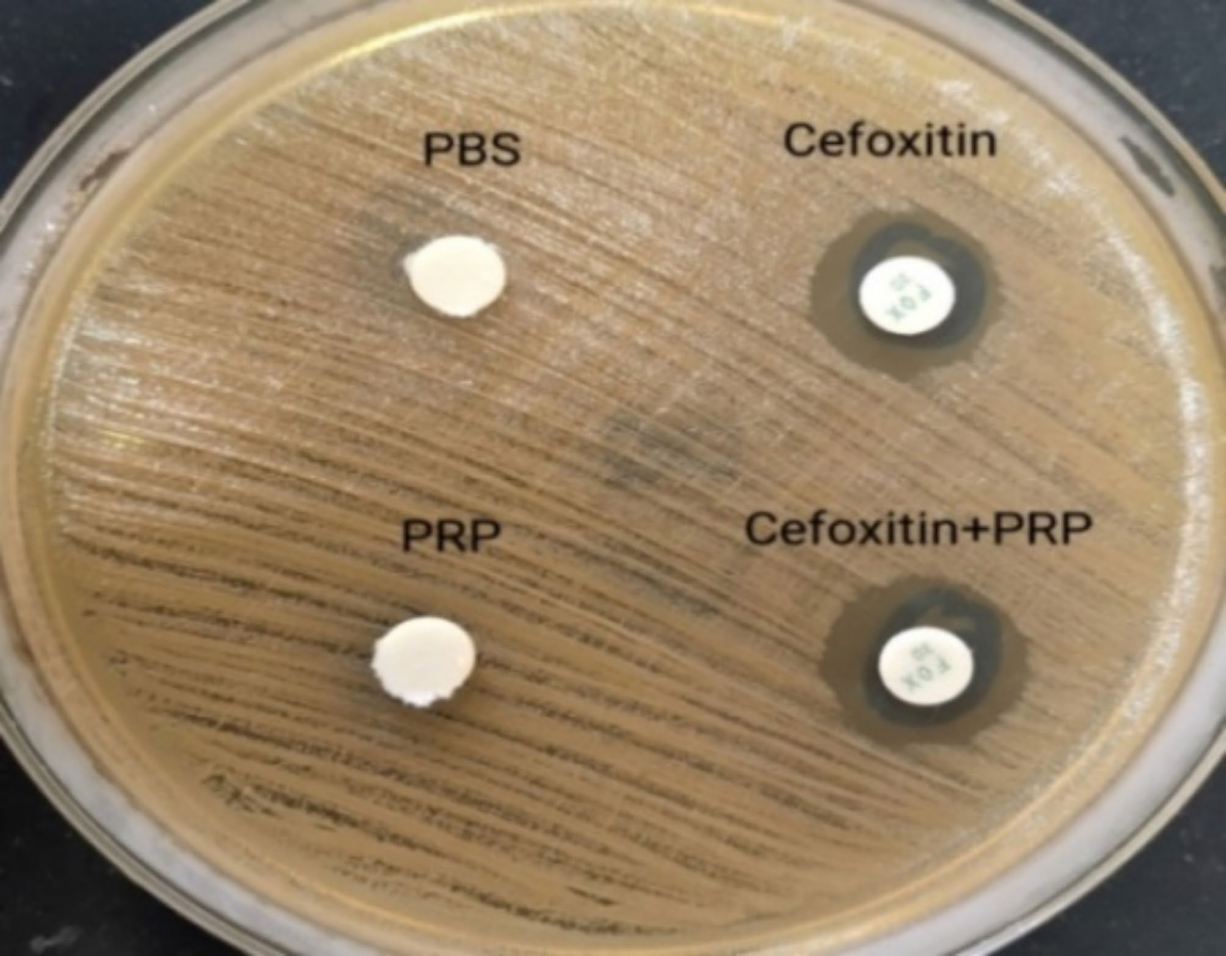
Antibacterial effect of PRP on *S. aureus* isolate via the disk diffusion method: empty discs were coated with one experimental PRP and an equal volume of PBS, as a control. These discs were then placed on inoculated Mueller-Hinton agar (MHA) plates. Standard 6 mm antibiotic discs of cefoxitin, alone and in combination with the experimental PRP, were also placed on the same MHA plates. No increase in the diameter of the inhibition zones was observed for the PRP-coated discs in comparison to the uncoated discs.

The average inhibition zone measured 6.00 mm (disc diameter) for non-activated PRP, activated PRP, and phosphate-buffered saline (PBS) against the examined isolates. No increase in the diameter of the inhibition zones was observed for the PRP-coated cefoxitin and ceftazidime discs in comparison to the uncoated antibiotic discs (Fig. 1).

#### Broth microdilution method

Using the broth microdilution method, all the studied MRSA, *K. pneumoniae*, and *P. aeruginosa* isolates were resistant to non-activated PRP. The activation of PRP by any of three methods (calcium gluconate, thrombin, or calcium gluconate plus thrombin) led to the formation of platelet gel, which precipitated at the bottom of the wells of microtiter plates. Therefore, the antibacterial activity of activated PRPs could not be evaluated. However, It was observed that wells containing suspensions of PRP (non-activated or activated) inoculated with *P. aeruginosa* presented yellow-green pigmentation compared with the dark-green pigmentation of those without PRP.

After 24 h of incubation of tested mixtures (PRP plus bacterial suspensions) that were plated onto tryptic soy agar (TSA) plates to determine the bactericidal activity of PRP, the non-activated and activated PRP did not show any bactericidal activity against the MRSA, *K. pneumoniae*, or *P. aeruginosa* isolates.

#### Checkerboard synergy testing

PPR-antibiotic combinations had synergistic effects on only 12.50 and 37.50% of MRSA and *P. aeruginosa* isolates, respectively. On the other hand, they had antagonistic activity against 37.50, 19.05, and 25.00% of MRSA, *K. pneumoniae*,* and P. aeruginosa* isolates, respectively. However, the outcome of the combination was indifferent to half of the MRSA (50.00%) and the majority of the *K. pneumoniae* (80.95%) isolates (Table 2).

**Table 2:** Interpretation of checkerboard synergy testing of prp‒antibiotic combinations against the 53 studied bacterial isolates, Alexandria, 2021–2022.

Studied bacterial Isolates	PRP-antibiotic combination*					
	Synergistic		Indifferent		Antagonistic	
	No.	%	No.	%	No.	%
MRSA^≠^(*n* = 16)	2	12.50	8	50.00	6	37.50
K.* pneumoniae*^†^(n = 21)	0	0.00	17	80.95	4	19.05
P.* aeruginosa*^†^(n = 16)	6	37.50	6	37.50	4	25.00
Total (*n *= 53)	8	15.09	31	58.49	14	26.42
Test of significance	χ^2^ = 12.964 _P_^MC^= 0.011°					

*Non-activated PRP.

≠PRP-cefoxitin combination against MRSA.

†: PRP-ceftazidime combination against *K. pneumoniae* or *P. aeruginosa*.

χ^2^: Pearson chi-square test.

_P_^MC^: Monte Carlo correction for the p value of the Pearson chi-square test.

°:* p*-value is significant at level < 0.05.

#### Time-kill assay

**Fig. 2:**
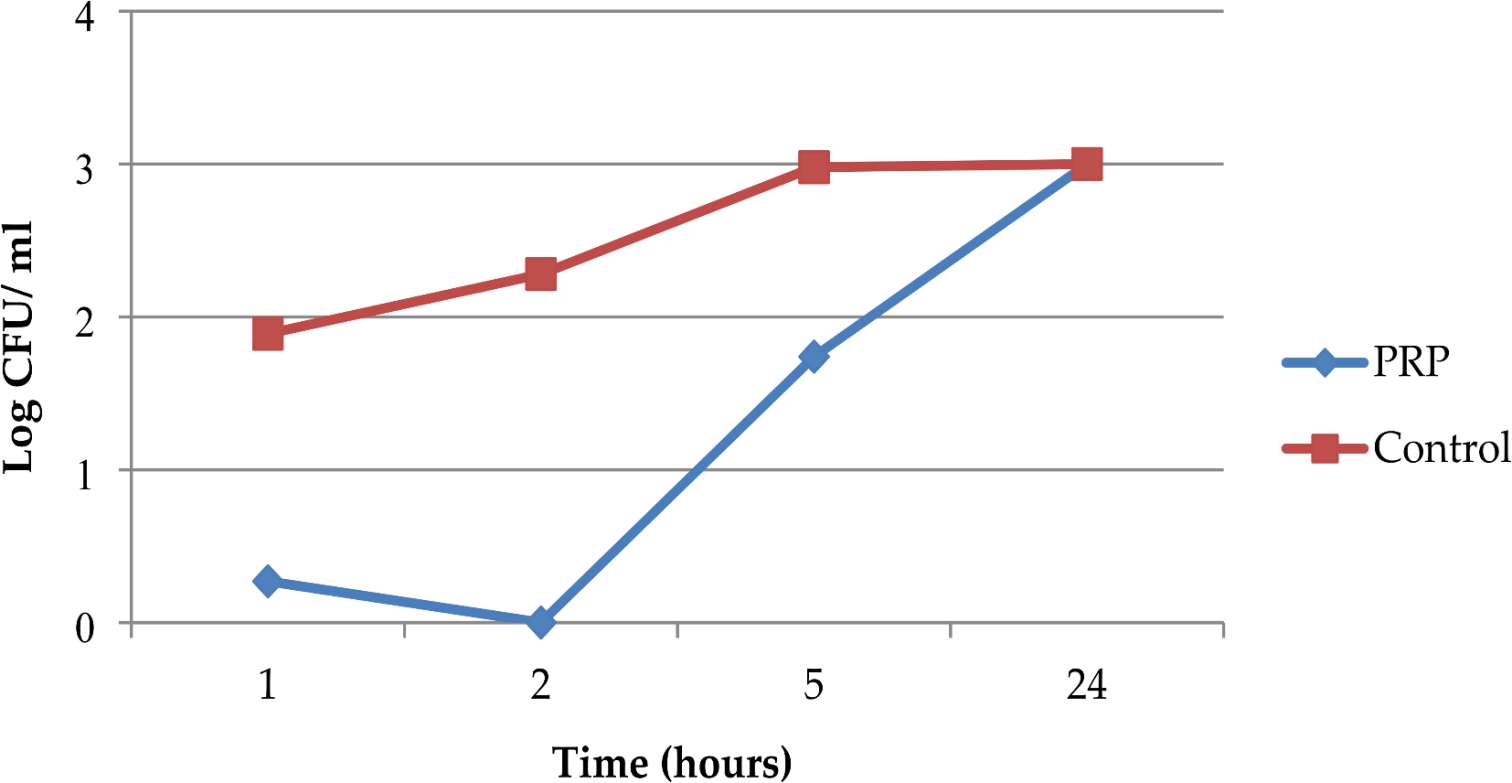
Time‒kill curve analysis of the 16 studied MRSA isolates in the presence of PRP, Alexandria, 2021–2022.

The mean colony counts for MRSA isolates in the PRP group were 1.87, 1.00, 54.81, and 1000.00, while in the control group, the counts were 77.93, 192.37, 946.00, and 1000.00 at 1, 2, 5, and 24 h, respectively. The antimicrobial effect of PRP peaked at the second hour of incubation, demonstrating a reduction of over 2 logs in colony count compared to the control group, and persisted until the fifth hour of incubation. Nonetheless, the effectiveness of the PPR diminished after the second hour of incubation. In comparison to the control group, the peak effectiveness of PRP inhibited approximately 99.50% of MRSA growth rate (Fig. 2).

**Fig. 3:**
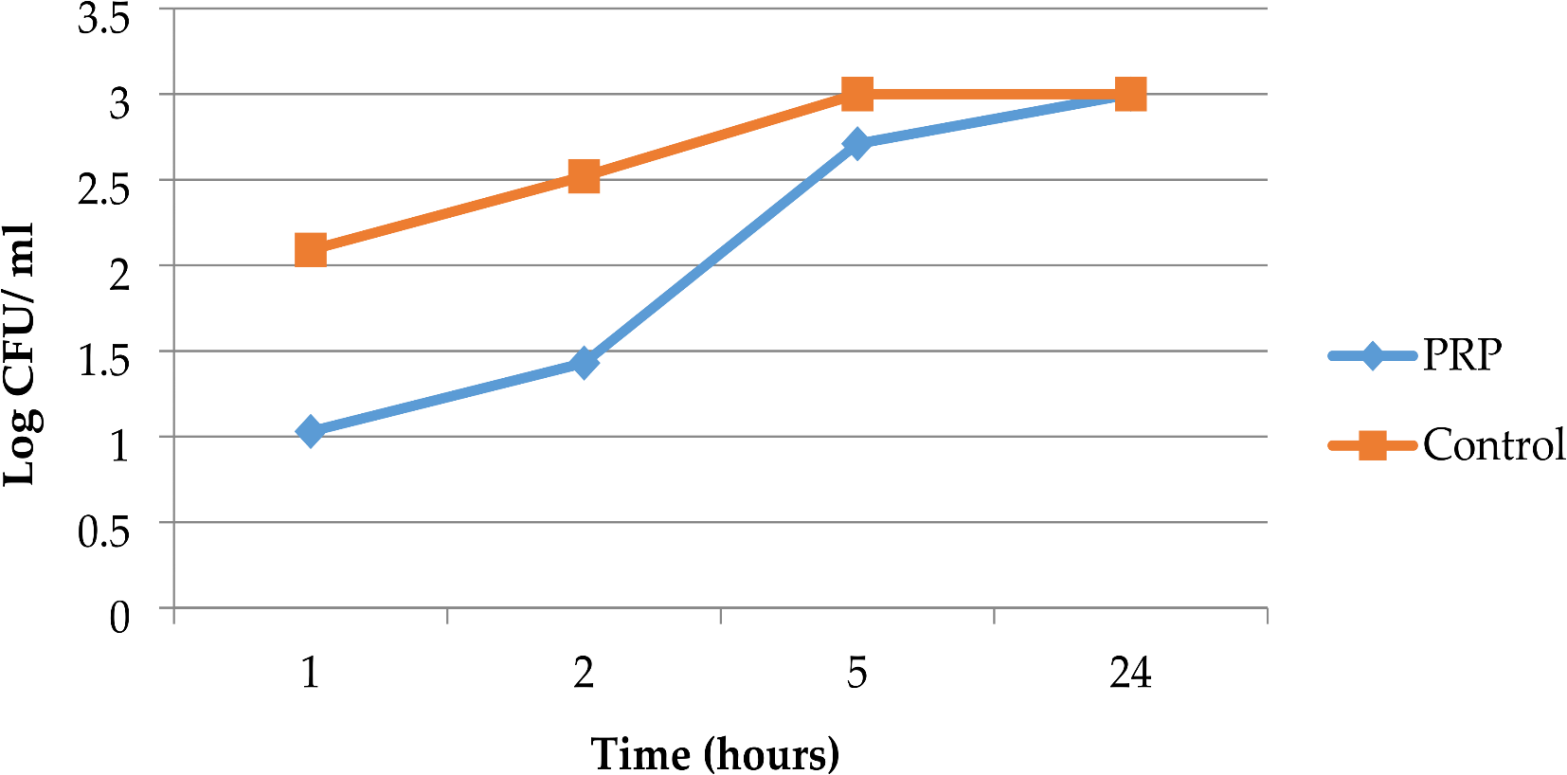
Time‒kill curve analysis of the 21 studied *K. pneumoniae* isolates in the presence of PRP, Alexandria, 2021–2022.

The mean colony counts for *K. pneumoniae* isolates in the PRP group were 10.66, 26.85, 516.04, and 1000.0 at 1, 2, 5, and 24 h, respectively. In the control group, the counts were 123.19, 332.52, 1000.00, and 1000.00 at the same time intervals. The antimicrobial effect of PRP peaked at one hour of incubation, demonstrating a one-log-fold reduction in colony count compared to the control group, and persisted until the fifth hour of incubation. Nonetheless, the effectiveness of the PPR diminished after the initial hour of incubation. The peak effectiveness of PRP inhibited approximately 91.35% of *K. pneumoniae* growth compared to the control group (Fig. 3).

**Fig. 4:**
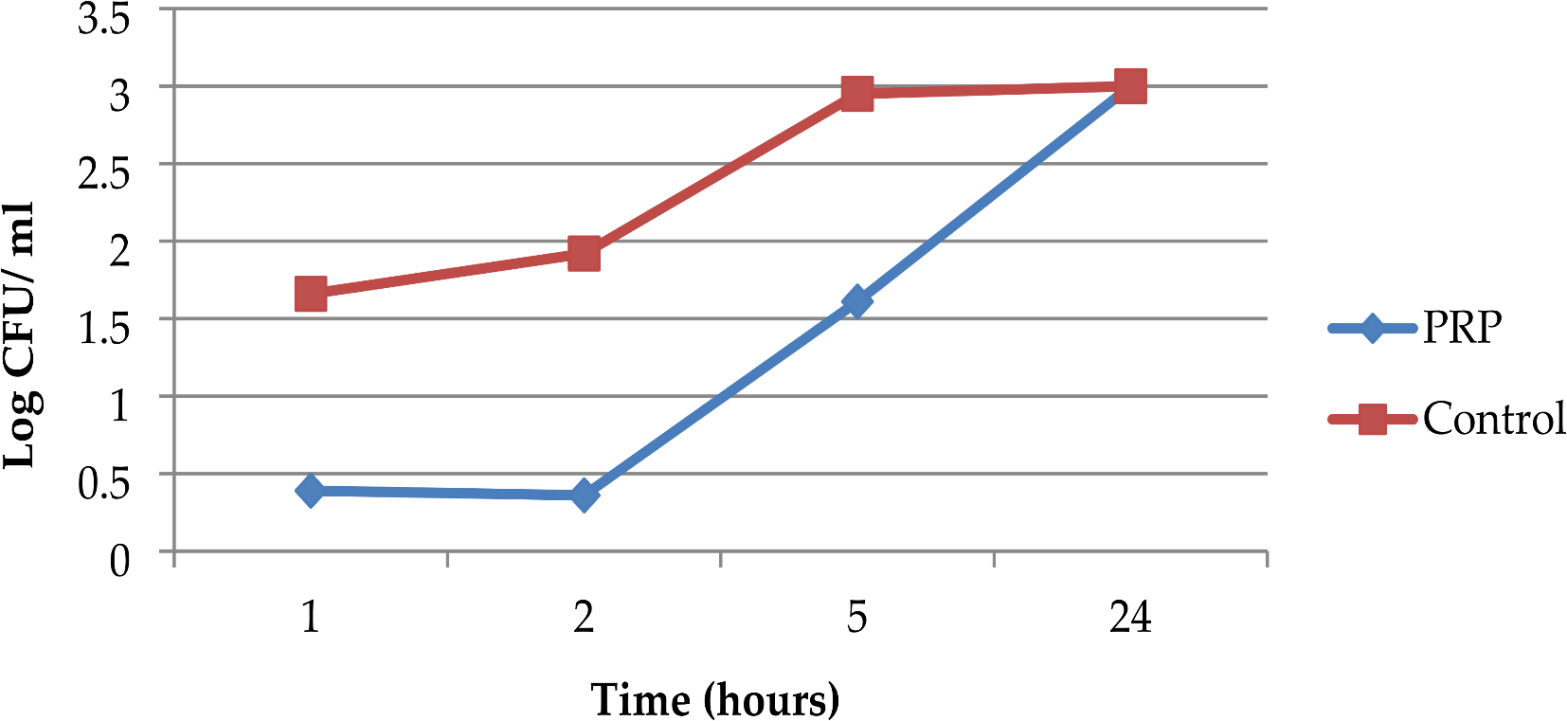
Time‒kill curve analysis of the 16 studied *P. aeruginosa* isolates in the presence of PRP, Alexandria, 2021–2022.

The mean colony counts for *P. aeruginosa* isolates in the PRP group were 2.43, 2.31, 41.06, and 1000.00, while in the control group, they were 46.18, 83.43, 884.37, and 1000.00 at 1, 2, 5, and 24 h, respectively. The antimicrobial effect of PRP peaked at the second hour of incubation, demonstrating an approximate one and a half-log reduction in colony count relative to the control group, and persisted until the fifth hour of incubation. Nonetheless, the effectiveness of the PPR diminished after the second hour of incubation. In comparison to the control group, the peak effectiveness of PRP inhibited approximately 97.23% of the growth rate of *P. aeruginosa* (Fig. 4).

These results revealed a highly statistically significant difference in the number of colonies of MRSA. *K. pneumoniae*, and *P. aeruginosa* isolates between the PRP and control groups after 1, 2, and 5 h of incubation (*p* < 0.0001). However, there was no statistically significant effect of PRP at 24 h of incubation (Table 3).

**Table 3:** Colony counts of the 53 bacterial isolates studied in the presence of PRP^†^ after 1, 2, 5, and 24 h of incubation on TSA, Alexandria, 2021–2022.

Studied bacterial isolates	Incubation time	Mean ± SD (CFU/ ml)		p*-*value of Mann-Whitney test
		PRP group	Control group	
MRSA (*n* = 16)	1 h	1.87 ± 2.94	77.93 ± 34.82	*p* < 0.0001*
	2 h	1.00 ± 2.90	192.37 ± 168.57	*p* < 0.0001*
	5 h	54.81 ± 56.80	946.00 ± 216.00	*p* < 0.0001*
	24 h	1000.00 ± 0.00^π^	1000.00 ± 0.00^π^	p = 1.000 “
K.* pneumoniae* (*n* = 21)	1 h	10.66 ± 15.80	123.19 ± 49.08	*p* < 0.0001*
	2 h	26.85 ± 42.36	332.52 ± 251.30	*p* < 0.0001*
	5 h	516.04 ± 475.35	1000.00 ± 0.00^π^	*p* < 0.0001*
	24 h	1000.00 ± 0.00^π^	1000.00 ± 0.00^π^	p = 1.000 “
P.* aeruginosa* (*n* = 16)	1 h	2.43 ± 186.00	46.18 ± 70.38	*p* < 0.0001*
	2 h	2.31 ± 2.96	83.43 ± 132.43	*p* < 0.0001*
	5 h	41.06 ± 41.56	884.37 ± 316.00	*p* < 0.0001*
	24 h	1000.00 ± 0.00^π^	1000.00 ± 0.00^π^	*p* = 1.000 “

†Activated PRP.

*Statistically significant (*p*-value is significant at < 0.05).

“Not statistically significant.

π: Colony counts over 1000 were recorded as 1000.

#### Determination of the antibiofilm activity of PRP

##### Detection of biofilm-forming bacterial isolates

**Fig. 5:**
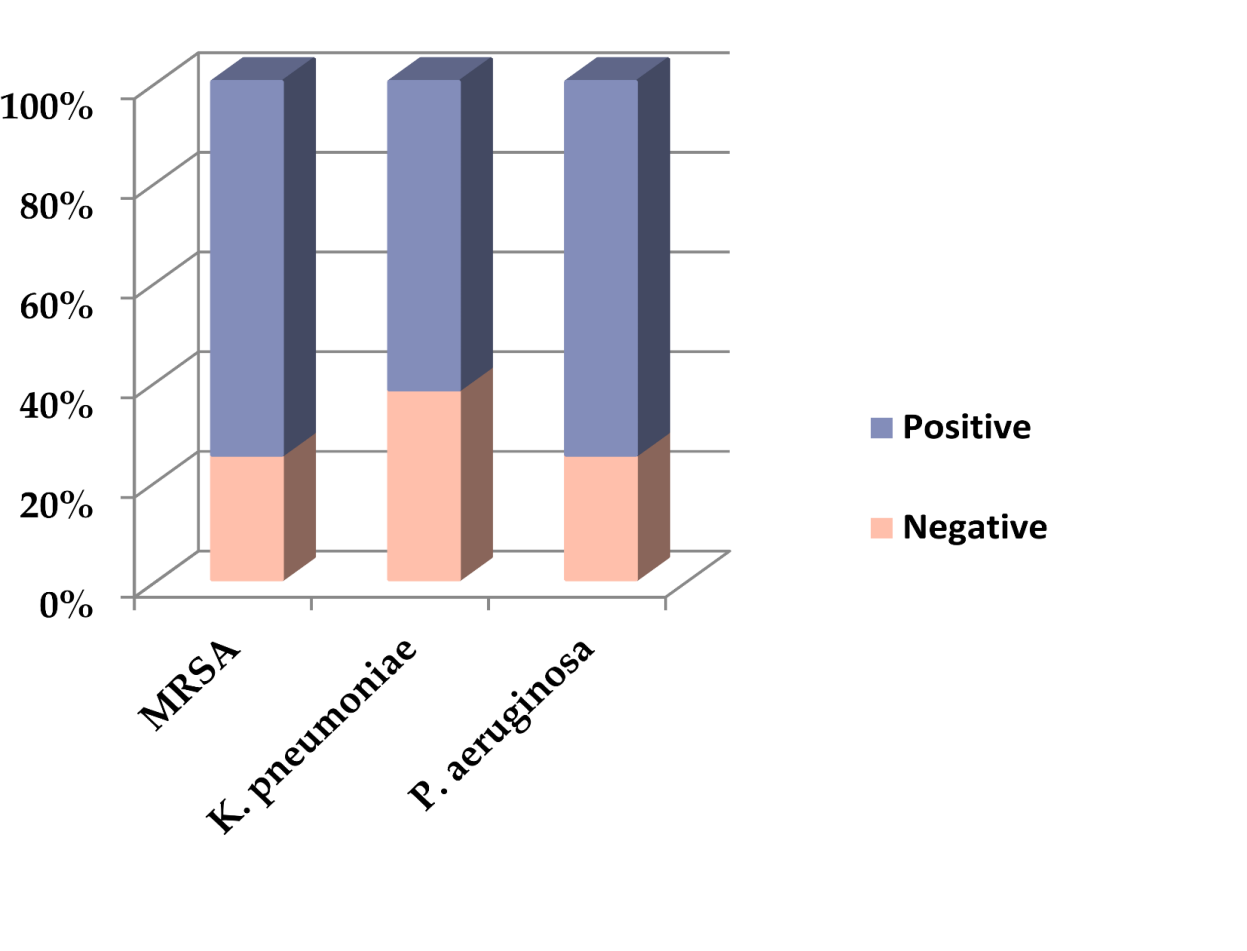
Biofilm-producing ability of the 53 bacterial isolates studied, Alexandria, 2021–2022.

Most of the studied isolates (*n* = 37, 69.81%) were biofilm producers. Moreover, among those biofilm-producing isolates, 11 (29.73%) were strong producers. Approximately 75.00% of the MRSA and *P. aeruginosa* isolates and 61.90% of the *K. pneumoniae* isolates were biofilm producers (Fig. 5; Table 4).

**Table 4:** Biofilm-producing ability of the 53 bacterial isolates studied, Alexandria, 2021–2022.

Studied bacterial isolates	Biofilm production							
	Negative		Weak		Moderate		Strong	
	No	%	No	%	No	%	No	%
MRSA (*n* = 16)	4	25	5	31.25	4	25	3	18.75
K.* pneumoniae* (*n* = 21)	8	38.09	5	23.81	4	19.05	4	19.05
P.* aeruginosa* (*n* = 16)	4	25	5	31.25	3	18.75	4	25
Total	16	30.19	15	28.30	11	20.75	11	20.75
Test of significanc	χ^2^ = 1.167 _P_^MC^= 0.997*							

χ^2^:Pearson chi-square test.

_P_^MC^: Monte Carlo correction for the p value of the Pearson chi-square test.

*: p value is significant at level < 0.05.

##### Biofilm inhibition and eradication assays

Both non-activated and activated PRP did not affect biofilm formation or established biofilms.

## Discussion

DFIs are among the most common complications of DM^24^. The emergence of resistance to commonly used antibiotics has become a threat among patients with DFIs and can lead to treatment failure with poor prognosis^25–27^. The development of alternative treatment methods that may help prevent or control DFIs could have substantial clinical, social and economic effects. According to some research, PRP is an effective antimicrobial agent that may be beneficial in the treatment of chronic wound or bone infections^15^. PRP has major advantages over conventional antibiotic treatment for DFIs, as it is less effective at inducing bacterial resistance. In addition, PRP not only reduces infections but also promotes wound healing.

Conflicting reports exist concerning the application of PRP in the management of infected wounds. Research indicates that plasma may serve as an effective medium for bacterial proliferation and may facilitate the entrapment of microorganisms within the platelet-fibrin matrix following platelet aggregation, potentially shielding pathogens from the effects of antibiotics or leukocytes^28,29^. Additional research indicates that PRP exhibits bacteriostatic and/or bactericidal properties against microorganisms^30–33^.

A standardized protocol for PRP preparation is currently lacking, which hinders the ability to compare efficacy across studies^15^. Amable et al. investigated fifteen distinct conditions, such as relative centrifugal force, centrifugation time, and temperature, to identify the optimal procedure for achieving the best platelet counts. The findings revealed an increase in platelet count ranging from 0.6 to 5.2-fold, contingent upon the specific conditions observed. This finding highlights that a crucial factor affecting PRP activity is the preparation process designed to achieve optimal platelet counts^34^. In our study, we utilized a PRP preparation protocol that resulted in an average 3.53-fold increase in platelet count compared to whole blood.

The role of leukocytes in PRP is controversial. Some evidence suggests that adding WBCs to PRP may boost its antimicrobial properties^35^. However, Anitua found that leukocytes did not significantly improve PRP’s antimicrobial properties^36^. Since white blood cells release proinflammatory proteases and metalloproteases, higher leukocyte concentration may worsen the inflammatory response^37^. Thus, we used a method to generate PRP with low leukocyte concentration.

In the present study, PRP activation was conducted immediately prior to application by incorporating 10% calcium gluconate, autologous thrombin, or a combination of both. An autologous platelet-rich gel (APG) made from whole blood contains PRP, calcium, and thrombin. Activated PRP releases GFs, antimicrobial proteins, and inflammatory cytokines. Clean DFUs have been treated with APG for years, improving ulcer healing^22,38–41^. Bovine thrombin was initially employed as an activating agent; however, the infrequent yet significant risk of coagulopathy due to antibody formation has limited its regular application. The use of calcium gluconate in the present study offers an alternative means of in vitro activation^42,43^.

In the current study, we evaluated the antibacterial efficacy of PRP against three MDR microorganisms isolated from infected DFUs, namely, MRSA, *K. pneumoniae*, and *P. aeruginosa*. The three bacterial species examined were selected because they represent the most common causes of DFIs.

To test the antibacterial effect of a substance, we can use different methods such as the disk diffusion method, broth dilution method, agar dilution method, or time‒kill assay^44^. We tested the in vitro antibacterial efficacy of PRP (with and without activation) via the Kirby–Bauer disk diffusion method. Unfortunately, none of our PRP preparations inhibited the growth of the studied MRSA, MDR *K. pneumoniae*, or *P. aeruginosa* isolates after 24 h of incubation. The mean value of the inhibition zones against all the tested isolates was only 6.00 mm (disc diameter) for both non-activated and activated PRP-coated empty discs. In addition, there was no increase in the diameter of the inhibition zones for the PRP-coated cefoxitin and ceftazidime discs compared with the uncoated antibiotic discs. Cetinkaya et al. found a 2 mm increase in PRP-coated empty disc diameter against *K. pneumoniae* and *P. aeruginosa* but no increase in MRSA inhibition zones^45^. In contrast, Bielecki et al. reported that PRP inhibited the growth of some MRSA strains via the disk diffusion method but had no effect on *K. pneumoniae* or *P. aeruginosa*. Moreover, PRP induced in vitro growth of *P. aeruginosa*^46^. In the previous study, PRP was prepared manually, resulting in a lower mean platelet count (228 × 109/L) compared to the present study. Cetinkaya et al. used an automated device to obtain PRP with concentrations seven times higher than the donor’s baseline platelet count. The median platelet count in PRP was 2208 × 109/L. These findings suggest a potential correlation between elevated platelet counts in PRP solutions and enhanced antimicrobial effects.

Owing to the failure of the disk diffusion method to demonstrate any antibacterial effect of PRP preparations, we tested the broth microdilution method, which in turn showed no antibacterial activity of non-activated PRP against the three tested bacterial species. Furthermore, the activation of PRP led to the formation of a gel that precipitated at the bottom of the wells of microtiter plates. Therefore, the bacteriostatic activity of activated PRPs could not be evaluated via the broth microdilution method. Hence, the bactericidal activity of the activated PRPs was evaluated by plating the tested mixtures onto TSA plates and counting the colonies. However, no bactericidal effect was detected.

Contrary to the findings of Attili et al., which indicated a decrease in the bacterial load of *P. aeruginosa* due to PRP, Edelblute et al. found no antibacterial efficacy of PRP against *P. aeruginosa*; however, notable bactericidal activity was detected against 40% of their *S. aureus* isolates using the broth microdilution technique^33,47^. This may be due to the fact that none of the *S. aureus* strains examined in their investigation were MRSA. Drago et al. indicated that PRP exhibited no efficacy against *P. aeruginosa*; however, it did provide growth suppression against other bacteria, including *Candida albicans*, *Streptococcus agalactiae*, *Streptococcus oralis*, and *Enterococcus faecalis*^11^.

Most *P. aeruginosa* strains produce one or more extracellular pigments, including pyoverdine (yellow–green), pyocyanin (blue–green), pyomelanin (brown–black), and pyorubrin (red–brown). In our study, wells containing suspensions of *P. aeruginosa* incubated with PRP presented yellow–green pigmentation compared with the dark–green pigmentation of those without PRP. These findings indicate that PRP led to the production of pyoverdine at the expense of pyocyanin. According to Abdelaziz et al., the synthesis of extracellular pigments is affected by the composition of incubation media, such as nitrogen and carbon sources. Hence, the production of pyoverdine in the present study might be attributed to the presence of organic nitrogen sources in PRP^48^.

Another trial was performed via checkerboard synergy testing to demonstrate the effect of PRP in combination with antibiotics. This method also had indifferent effects on most of the tested isolates (58.49%). Although still non bacteriostatic, it had a synergistic effect on only 12.50% of the MRSA isolates when combined with cefoxitin and on 37.50% of the *P. aeruginosa* isolates when combined with ceftazidime. However, it showed antagonistic activity against 26.42% of the tested isolates. No further studies that used checkerboard synergy testing of PRP with antibiotics were found in the literature. However, Cetinkaya et al. demonstrated that PRP exhibited a synergistic effect on MRSA when used in conjunction with vancomycin in a rat model of surgical wound infection^49^.

The last trial was performed via a time-kill assay. Finally, this method showed a highly statistically significant antimicrobial effect of PRG (activated PRP) against the three tested MDR strains compared with the controls after 1, 2, and 5 h of incubation. The peak point of effectiveness differed according to the studied microorganism. The efficiency of the PPR decreased after the second hour of incubation for MRSA and MDR *P. aeruginosa*, and after the first hour for MDR *K. pneumoniae*. PRP had a peak effectiveness of about 99.50%, 91.35%, and 97.23% against the growth rates of MRSA, *MDR K. pneumoniae*, and MDR *P. aeruginosa*, respectively. PRP’s antimicrobial impact lasted up to 5 h. When all of the tested strains were examined after 24 h of incubation, they all demonstrated regrowth and the elimination of the inhibitory effect of PRP, as compared to the controls. Cetinkaya et al. also discovered that PRP had antimicrobial effects on MRSA, ESBL-producing *K. pneumoniae*, and carbapenem-resistant *P. aeruginosa*; however, the effects were statistically significant only on MRSA and *P. aeruginosa*, and only in the first hour of their study, which is consistent with the findings of the current study^45^. Moojen et al. found similar findings, stating that PRP had a strong antibacterial impact that was limited to the first hour after application and could maintain a proportional reduction in bacteria of roughly 99% compared to the control for up to 8 h^50^. According to Hasan et al., the maximal antibacterial activity of PRP against *S. aureus* was limited to the first hour after application, and the antimicrobial effect peaked at 4 h with an 84.2% reduction in bacterial count. This rate dropped to 76.3% after 24 h^17^.

Compared with planktonic bacteria, bacterial biofilms are known to exhibit significantly greater resistance to antimicrobial agents. Therefore, they demand substantially higher concentrations of antimicrobial factors to exert any effect. This heightened resistance is due to the ability of biofilm-producing bacteria to shield themselves within the extracellular polymeric substance (EPS). Antimicrobial agents released by substances find it challenging to penetrate biofilm cultures because of their diffusion-limited transport through biofilm EPS. As these agents pass through, they become diluted, making it difficult for them to reach bacterial colonies^51,52^.

In our study, 69.81% of the studied isolates were biofilm producers, and PRP had no effect on biofilm formation or established biofilms. Similar findings were reported by Smith et al.^53^. In contrast, Gilbertie et al. found that PPR and amikacin had synergistic efficacy against aminoglycoside-tolerant biofilm formations, with stronger activity against gram-positive bacteria^54^. This could be due to a specific “window of opportunity” when a treatment can effectively target biofilms, occurring between the initial adhesion and irreversible binding of bacteria. During this phase, bacteria are vulnerable, but once a biofilm is formed, it becomes resistant to penetration. Consequently, topical PRP application appears ineffective against well-established biofilms. In clinical settings, PRP might be more effective when combined with antibiotic therapy against these firmly established colonies^55^.

In vivo experiments yielded better findings for PRP’s antibacterial action than in vitro investigations. Sun et al. successfully eliminated the infection and achieved wound closure using autologous PG and negative pressure wound therapy in a case involving a diabetic patient and an infected amputation caused by MDR *Acinetobacter baumannii* that remained unresolved despite extended antibiotic treatments^56^. In a rabbit model of implant-associated spinal infection, Li et al. found that PRP treatment significantly reduced bacterial colonies in bone samples and improved bone healing post-operatively^57^. However, our study indicated that the bacterial inhibition observed, when present, was temporary, suggesting a possible depletion of plasma antibacterial factors. This could be attributed to bacteria being less susceptible to direct topical application of PRP without an immune response providing additional antimicrobial support. These findings have practical implications for the clinical use of blood-derived biomaterials. Particularly noteworthy is the potential exploration of whether regular PRP application to wounds could mitigate the risk of bacterial regrowth and infections. As a result, more rigorous clinical evidence is required to justify the use of PRP in DFIs. Future large-scale prospective randomized controlled clinical trials should be conducted to develop uniform guidelines for the indications, contraindications, and particular methods for PRP use in clinical settings.

In conclusion, only activated PRP effectively inhibited microbial growth, indicating that coagulation activation is a crucial step. Bacterial growth inhibition by PRP was apparent immediately after activation; however, it was temporary and persisted for up to 5 h, varying by bacterial strain, indicating differential susceptibility to or consumption of the antibacterial factors present in the PRP suspension. Consequently, PRP can function synergistically with antibiotics and serve as an additional therapy for infections. Variations in study designs, including PRP preparation, platelet activation, bacterial kind, and the antibiotic resistance profile of bacteria, may account for the disparate results. Nevertheless, the majority of in vitro and in vivo investigations have demonstrated that there are no contraindications for the application of PRP in infected wounds.

### Methods

An in vitro experimental study was conducted from April 2021 to May 2022 at the HIPH and the Clinical Pathology Laboratory at AMUH. The study proposal was approved by the Ethics Committee at the HIPH, Alexandria University, Egypt. Informed consent was obrained from each participant in this study. All methods were performed in accordance with the relevant guidelines and regulations for ethics approval and consent to participate.

**Fig. 6:**
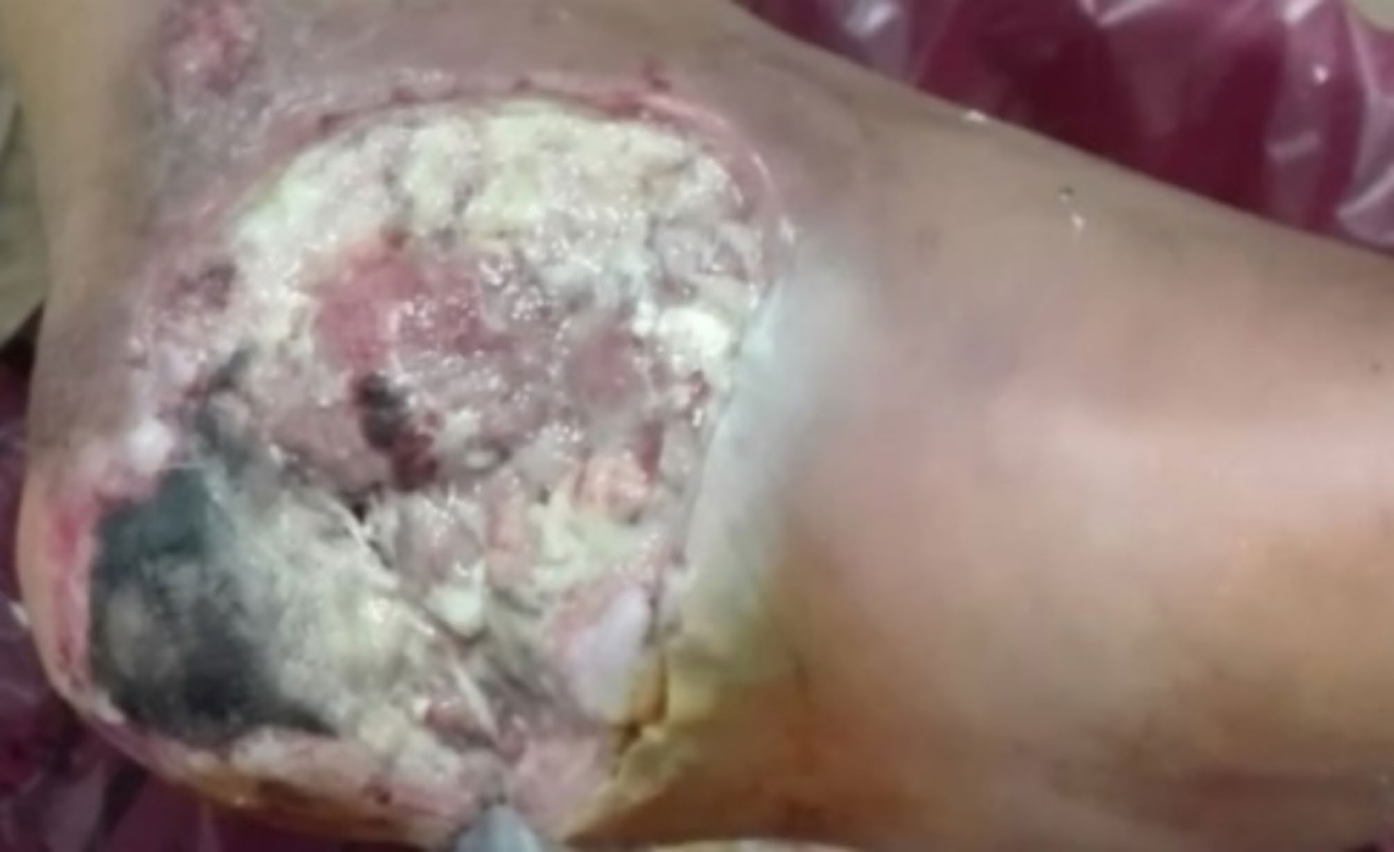
Diabetic foot ulcer.

To collect the isolates required for the study, a total of 78 swab samples were randomly collected from clinically suspected infected DF lesions of patients admitted to the Vascular Surgery and Diabetic Foot Unit at the Surgery Department in AMUH (Fig. 6). Bacterial isolates other than MRSA, MDR *K. pneumoniae*, and MDR *P. aeruginosa* were excluded from the PRP experimental study.

### Sampling and processing of samples

No antimicrobial agent or antiseptic agent was introduced into diabetic foot wounds before samples were collected. Swab samples were collected via Levine’s technique^58^. The swabs were then replaced into their containers and transported directly without refrigeration to the Microbiology Laboratory at the HIPH.

The collected swabs were inoculated onto blood agar and MacConkey agar plates. The plates were incubated in aerobic conditions at 37 °C for 24 to 48 h. All isolated colonies on the blood and MacConkey agar plates were identified following conventional microbiological procedures^59^. All isolates of *S. aureus*, *K. pneumoniae*, and *P. aeruginosa* underwent antibiotic susceptibility testing using the Kirby-Bauer disk diffusion method on MHA plates^60^. Isolates of *S. aureus* exhibiting resistance to cefoxitin (30 µg) were classified as MRSA^61^. *K. pneumoniae* and *P. aeruginosa* isolates exhibiting resistance to at least one agent across three or more antibiotic classes were classified as MDR^62^.

**Fig. 7:**
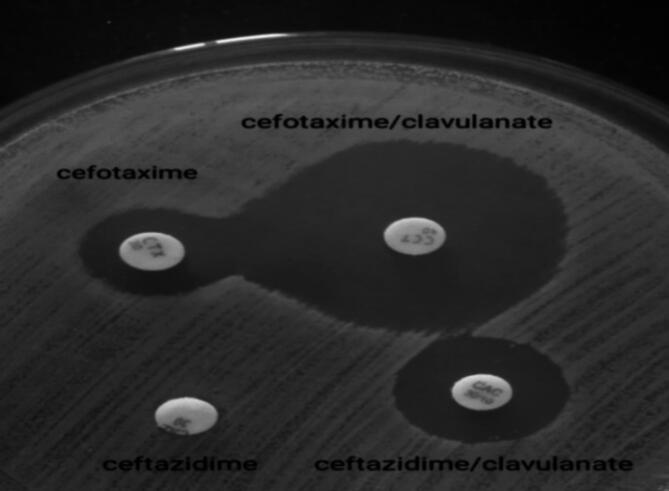
ESBL-producing *K. pneumoniae* isolate on MHA: a ≥ 5 mm increase in the zone diameter was observed for either antimicrobial agent tested in combination with clavulanate versus the zone diameter of the agent when tested alone.

**Fig. 8:**
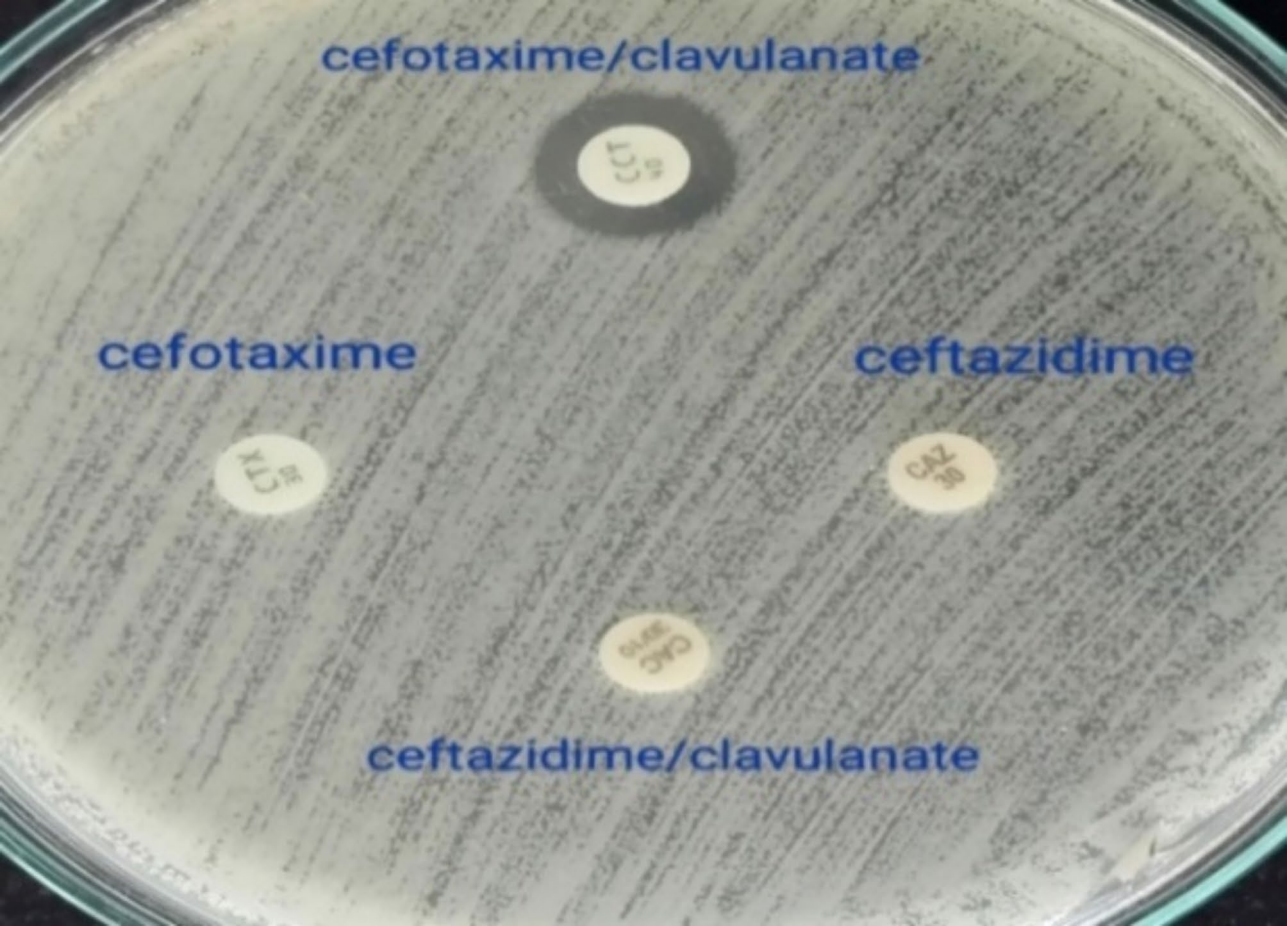
Non-ESBL-producing *K. pneumoniae* isolate on MHA.

All MDR *K. pneumoniae* isolates were tested for ESBL production, and bacterial suspensions were prepared via the direct colony suspension method. The bacterial suspensions were subsequently inoculated onto MHA plates. Subsequently, cefotaxime and ceftazidime discs, both individually and in combination with clavulanate (cefotaxime-clavulanate 30/10 µg and ceftazidime-clavulanate 30/10 µg), were placed on each plate, which were then incubated at 35–37 °C for a duration of 16–18 h. Positive ESBL production was noted when a > 5 mm increase in the zone diameter occurred for either antimicrobial agent tested when combined with clavulanate compared to the zone diameter of the agent tested individually (Figs. 7 and 8)^61^.

**Fig. 9:**
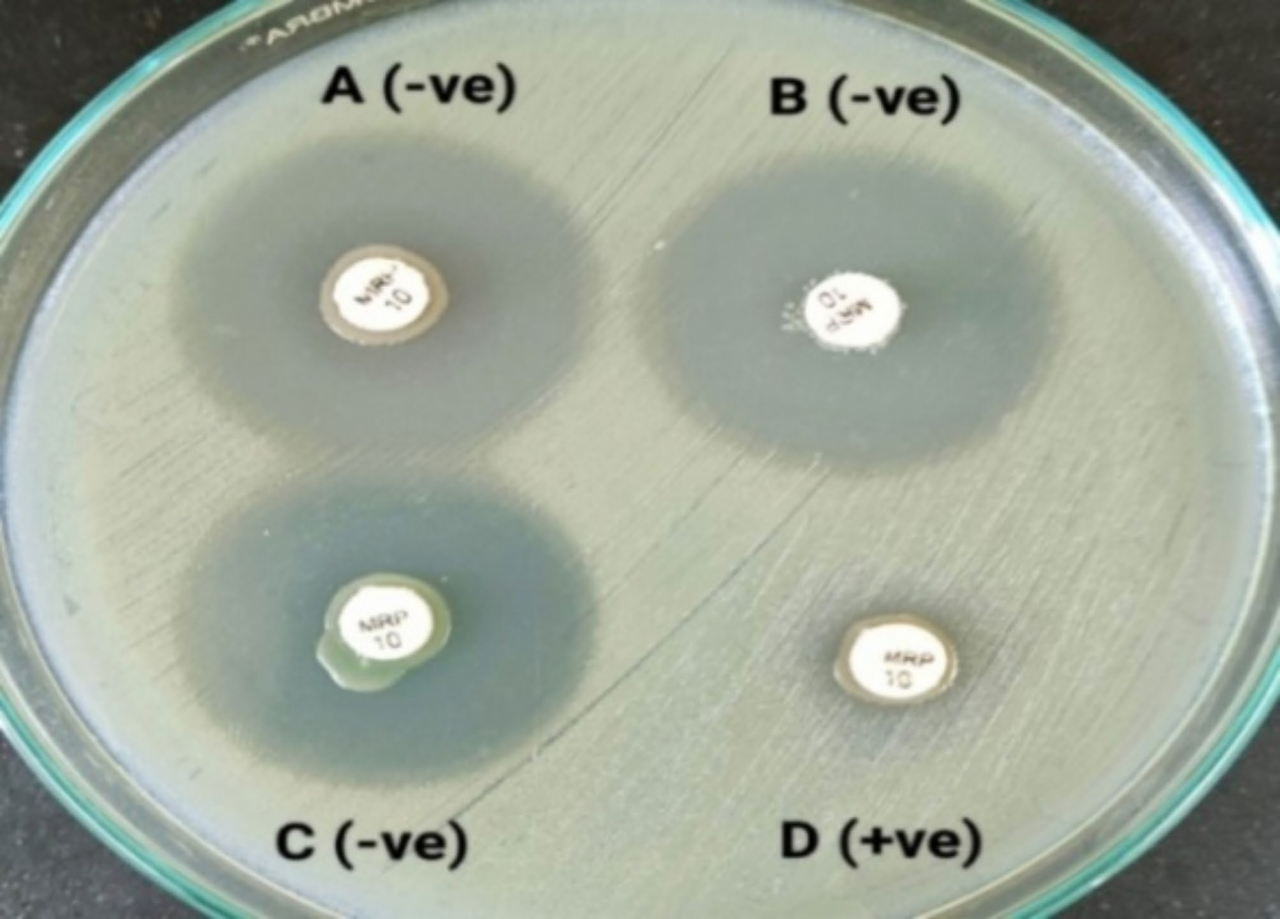
Testing of *P. aeruginosa* isolates for carbapenemase production by mCIM: (**A**–**C**) A zone diameter of ≥ 19 mm was considered a negative result (i.e., no carbapenemase production was detected); (**D**) A zone diameter of 6–15 mm or the presence of pinpoint colonies within a 16–18 mm zone was considered a positive result (i.e., carbapenemase production detected).

All MDR *P. aeruginosa* isolates were evaluated for carbapenemase production using the modified carbapenem inactivation method (mCIM), as per the guidelines of the Clinical and Laboratory Standards Institute (CLSI) (Fig. 9)^61^.

### Determination of the antibacterial activity of PRP

#### PRP preparation

##### Blood collection

For PRP preparation, 8–16 mL of venous blood samples were collected from each of 16 adult volunteers into sterile tubes containing the anticoagulant citrate dextrose-adenine (ACD-A). All the volunteers were predominantly in good health. The exclusion criteria included the administration of antimicrobials and/or anti-inflammatory drugs within the preceding 10 days, smoking, pregnancy, systemic diseases, infections, hemoglobin levels below 10 g/dL, and/or platelet counts of 100 × 10^3/µL or lower^12,63^.

##### Procedure of PRP preparation

**Fig. 10:**
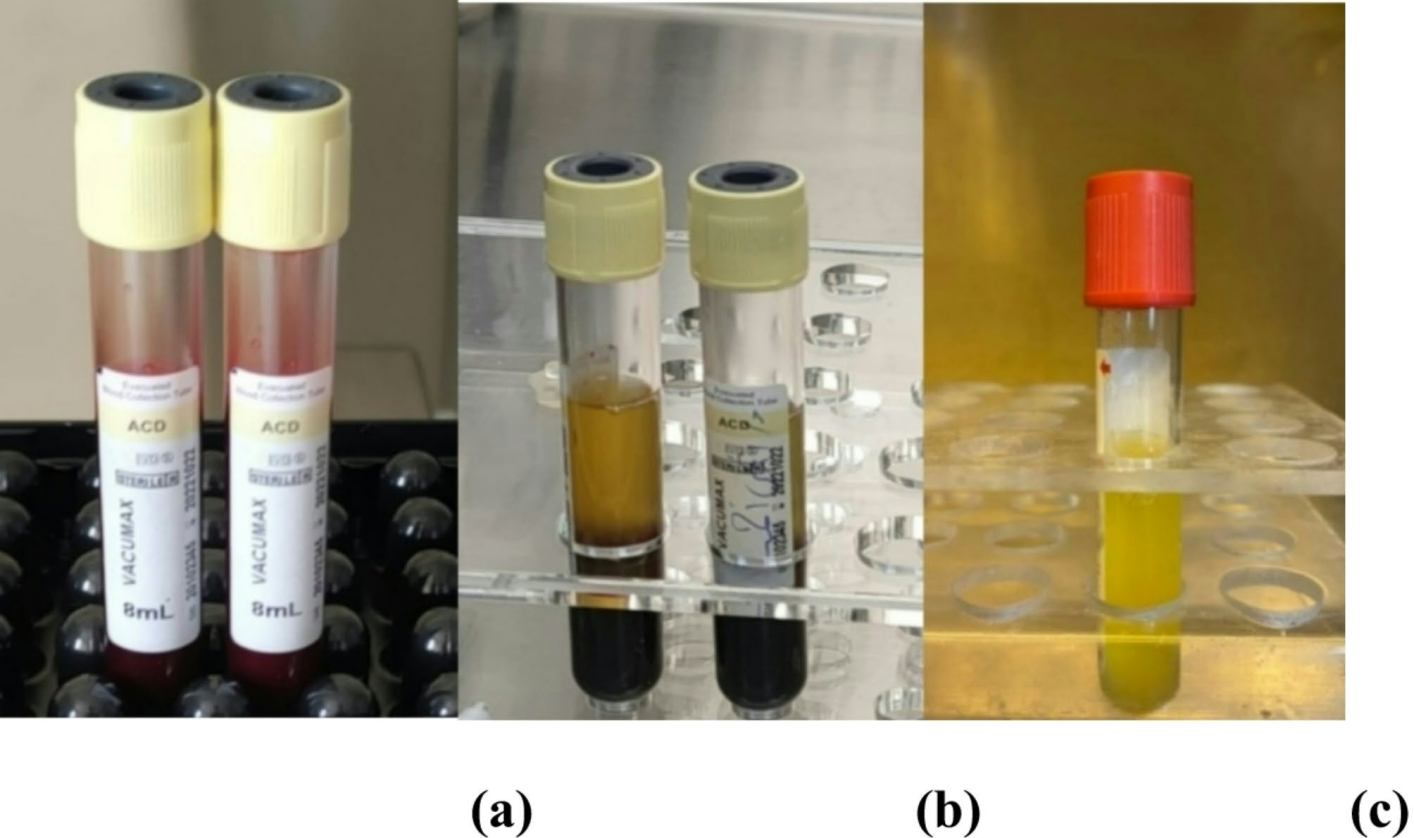
PRP preparation: (**a**) Venous blood collected in ACD-A tubes; (**b**) Separation of the blood into three layers after the first centrifugation (soft spin); (**c**) Prepared PRP (3 ml).

- Whole blood in the ACD-A tubes was centrifuged at 200–250 × g (1100–1200 rpm) for 10 min (Hettich, Germany). This initial centrifugation, termed ‘soft spin’, facilitates the separation of blood into three distinct layers: a bottom layer of red blood cells (RBCs) comprising 55% of the total volume, a top acellular plasma layer known as platelet-poor plasma (PPP) accounting for 40% of the total volume, and an intermediate layer referred to as the ‘buffy coat’, which contains PRP and constitutes 5% of the total volume.
- The PPP, PRP and some RBCs, specifically the upper two layers and a negligible ‘unavoidable’ quantity from the bottom layer, were placed into a separate tube devoid of anticoagulant.
- This tube was subjected to a secondary centrifugation at 300 × g (1300 rpm) for 15 min, referred to as the ‘hard spin’. This enabled the platelets (PRP) to accumulate at the tube’s base with minimal RBCs, whereas PPP constituted 80% of the volume at the top.
- The majority of the supernatant PPP was extracted using a syringe, and the platelet pellet was reconstituted in a minimal volume of PPP (1.5 ml) using vortexing (Nickel Electro LTD, England)^15,64,65^. The produced plasma was identified as the PRP utilized in the study (Fig. 10).

##### Determination of platelet and leukocyte counts

The count of WBCs and platelets in the PRP for each participant was assessed using a complete blood cell analyzer (Abbott, USA). The outcomes were then compared with the whole blood results of the same volunteer to assess the efficacy of PRP preparation.

##### Preparation of autologous thrombin

For thrombin preparation, approximately 4 ml of whole blood from each donor was drawn into a serum clot activation tube. The serum clot activation tubes were kept at room temperature for 10 min until clotting. The tubes were subsequently centrifuged at 3000 ×*g* for 3 min. The top layer (supernatant) was considered thrombin and was transferred to a new tube. This thrombin solution was subsequently used for PRP activation^45,66^.

##### PRP activation

Platelet activation was performed immediately before the use of PRP by adding the following:

- A 10% calcium gluconate at a ratio of 1:5 (0.6 ml of calcium gluconate to each 3 ml of PRP).

Or

- Autologous thrombin at a ratio of 1:4 (0.75 ml of autologous thrombin to each 3 ml of PRP).

Or

- A mixture of autologous thrombin and 10% calcium gluconate at a ratio of 2:1:8 (0.8 ml of autologous thrombin and 0.4 ml of 10% calcium gluconate to each 3 ml of PRP) to produce a platelet-rich gel^17,45^.

The experimental samples of PRP were divided into four types:

- Type 1: non-activated PRP.
- Type 2: calcium gluconate-activated PRP.
- Type 3: thrombin-activated PRP.
- Type 4: calcium gluconate with thrombin-activated PRP.

##### Kirby–Bauer disk diffusion method

The antibacterial activity of PRP was assessed using the Kirby–Bauer disk diffusion method on MHA plates^45^. Using sterile micropipettes, empty discs were coated with one of the following: 10 µL of the experimental PRP or an equal volume of PBS, which was used as a control. The discs were subsequently placed on inoculated MHA plates. Standard 6 mm antibiotic discs of cefoxitin, as well as others coated with the experimental PRP, were placed on the same MHA plates using separate micropipettes for MRSA testing. For MDR *K. pneumoniae* and MDR *P. aeruginosa*, one antibiotic from each class to which the tested strain exhibited resistance was applied, both individually and in combination with the experimental PRP, on MHA plates. The test was conducted separately for each of the four PRP types.

The test was conducted multiple times utilizing varying volumes of PRP: 20, 50, and 100 µL. Each type of PRP was tested through direct inoculation onto the media surface using a micropipette, ensuring contact with the microorganisms to minimize variables that could affect their antibacterial actions^47^. The plates were incubated in an inverted position at a temperature range of 35 to 37 °C for duration of 16 to 18 h. The diameters of the inhibition zones were measured using a ruler, and the antimicrobial activity of PRP was evaluated by comparing the zone diameter of the antibiotic disc tested in combination with the experimental PRP to the zone diameter of the disc tested independently (Fig. 2)^45,47^.

##### Broth microdilution method

The antibacterial effect of PRP was assessed via broth inhibition via the microtiter method^67^. The assay was performed as follows:

1. Fifty microlitres of each of the four types of PRP (a non-activated type and 3 activated types**)** were added to each of 4 wells (for each tested isolate).
2. Fifty microlitres of bacterial suspension of the tested isolate (1** × **10^6^ CFU/mL) were inoculated into each well containing different types of PRP.
3. The following controls were used: sterility control (100 µL of Mueller–Hinton broth (MHB)), growth control (50 µL of MHB with 50 µL of bacterial suspension), and PRP control (50 MHB with 50 µL of PRP).
4. The microtiter plate was incubated at 37 °C for duration of 16 to 20 h.
5. After incubation, the antibacterial effect of PRP was evaluated by observing the inhibition of visible growth of bacteria.
6. The observed turbidity or sediment (button size) indicated that there was no inhibition of visible growth of bacteria.
7. Approximately 100 µL from each tested mixture (PRP plus bacterial suspension) and the growth control were plated onto TSA plates and incubated at 37 °C for 16 to 20 h.
8. A comparison between the colony counts of the tested mixtures and those of the growth controls on TSA plates was used to determine the bactericidal activity of PRP.

##### Checkerboard synergy testing

The two-dimensional microdilution method in a 96-well microtiter plate was used to evaluate the antibacterial activity of PRP in combination with antibiotics against resistant isolates. The methodology for checkerboard synergy testing was adapted from previous studies but with some modifications^68,69^.

**Fig. 11:**
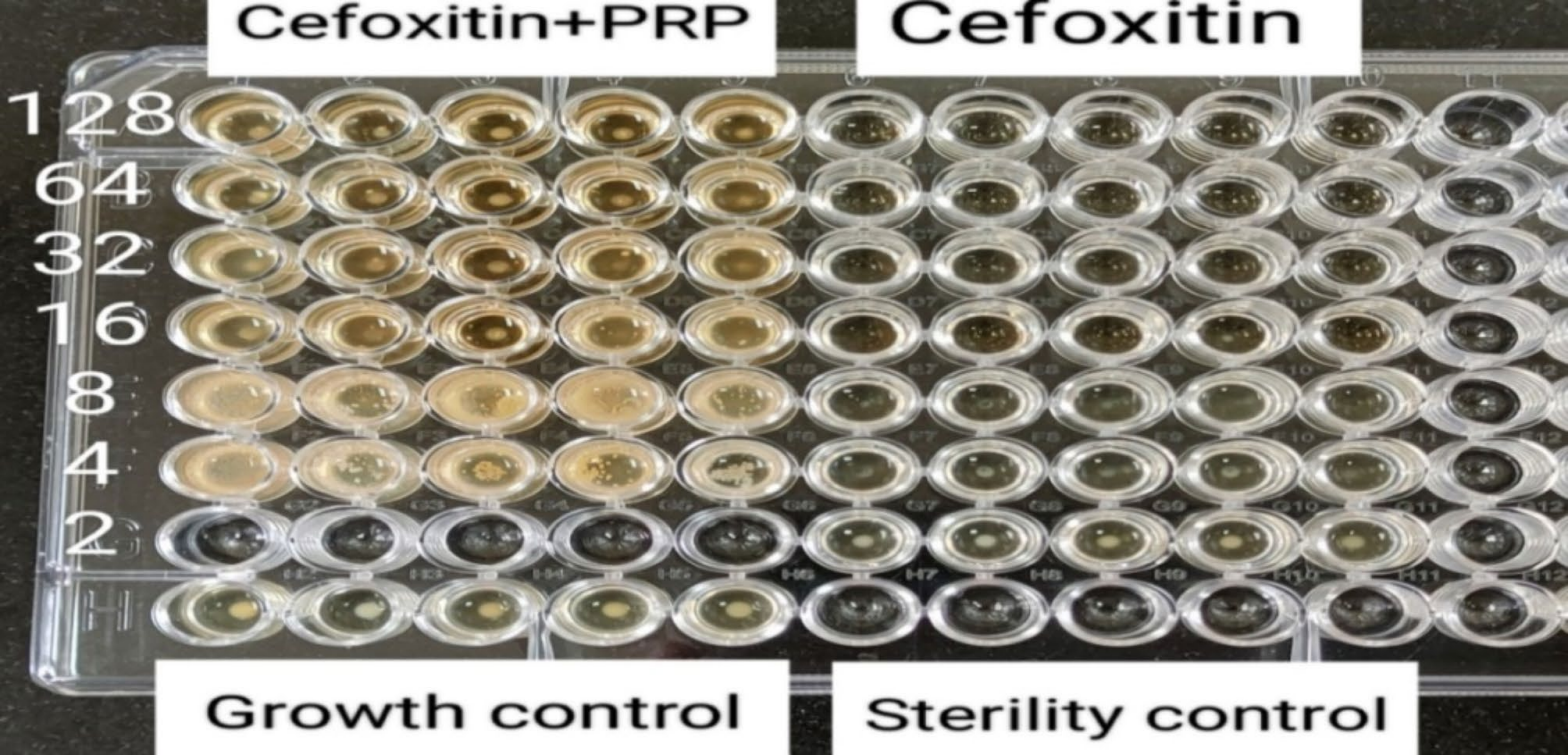
Checkerboard synergy testing of PRP and cefoxitin against MRSA isolates: each column from 1–5 contains different bacterial isolates with cefoxitin and PRP, whereas columns from 6–10 contain the same isolates present in columns 1–5 with cefoxitin and without PRP.

**Fig. 12:**
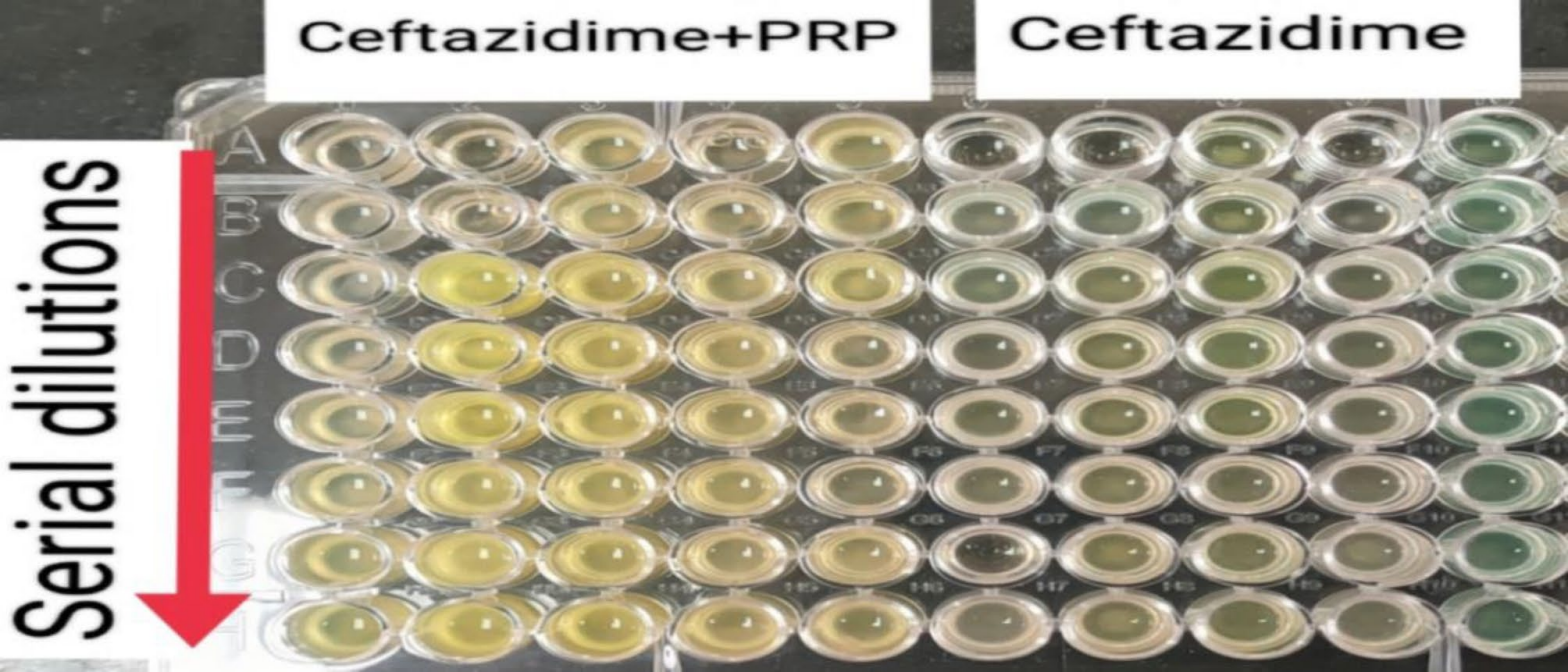
Checkerboard synergy testing of PRP and ceftazidime against *P. aeruginosa* isolates: each column from 1–5 contains different bacterial isolates with ceftazidime and PRP, whereas columns from 610 contain the same isolates present in columns 1–5 with ceftazidime and without PRP.

One antibiotic agent to which the bacterial isolates were resistant was tested. Cefoxitin sodium was tested against MRSA isolates, whereas ceftazidime pentahydrate was tested against *K. pneumoniae* and *P. aeruginosa* isolates. The minimal inhibitory concentration (MIC) was defined as the lowest antibiotic concentration that inhibited visible growth of the tested isolate, as observed with the naked eye, as shown in Figs. 11 and 12^67^. The experiment was conducted in duplicate for each isolate tested, and if the two MICs varied by more than two wells, the assay was repeated. The MIC of each antibiotic was assessed individually and in combination with PRP.

The combination of PRP with antibiotics was considered synergistic when the MIC of an antibiotic in combination with PRP was lower than the MIC of the antibiotic independently, indifferent when the MIC of an antibiotic in combination with PRP was the same as the MIC of the antibiotic independently, and antagonistic when the MIC of an antibiotic in combination with PRP was higher than the MIC of the antibiotic independently.

##### Time-kill assay

A time–kill assay was conducted to test the antibacterial effect of activated PRP against MRSA, *K. pneumoniae*, and *P. aeruginosa* isolates, where viable bacterial counts were determined for 10 µL aliquots taken from PRP and control tubes at 1, 2, 5, and 24 h^70^. The bacterial growth rate in the PRP tubes was compared with the simultaneous bacterial growth rate in the control tubes. The peak point of effectiveness for PRP against each bacterium was defined as the duration of assessment in the time-kill assay that exhibited the highest rate of bacterial growth inhibition.

##### Time-kill test procedure

- From freshly grown blood agar cultures, 3 to 5 colonies with a single morphology were touched lightly with a sterile wire loop and suspended in a tube containing 5 mL of prewarmed MHB.
- The bacterial suspension was incubated in a shaker incubator (150 rpm) at 37 °C until turbidity was reached.
- The turbidity of the actively proliferating broth culture in the exponential growth phase was calibrated to achieve a visual comparison with 0.5 McFarland turbidity standards (about 1 × 10^8 CFU/mL).
- Within 15 min of preparation, the adjusted suspension was diluted to 1:100 by adding 100 µL of the adjusted suspension to 9.9 mL of MHB, resulting in approximately 1 × 10^6^ CFU/mL.
- The adjusted dilution (containing 1 × 10^6^ CFU/mL) was further diluted by a factor of 1:10 by adding 100 µl of bacterial suspension to 900 µL of sterile MHB, resulting in a bacterial suspension containing about 1 × 10^5^ CFU/mL.
- One hundred microliters of each bacterial isolate, with controlled colony counts of 1 × 10^5 CFU/mL, were placed into sample tubes. Subsequently, 700 µL of MHB, 160 µL of PRP, and 40 µL of platelet activator (comprising autologous thrombin and 10% calcium gluconate in a 2:1 ratio) were introduced into the tubes, achieving a bacterial concentration of 1 × 10^4 CFU/mL.
- The tubes were placed in a shaker incubator at 37 °C.
- After 1, 2, 5 and 24 h of incubation, the tubes were vortexed, and 10 µL of each mixture was added to 90 µL of sterile saline in a sterile Eppendorf tube via a sterile pipette.
- The mixtures were incubated into TSA plates and incubated at 37 °C for 24 h.
- The same processes employed for the PRP tubes were applied to the control tubes, utilizing sterile saline in place of PRP.

##### Colony counts

**Fig. 13:**
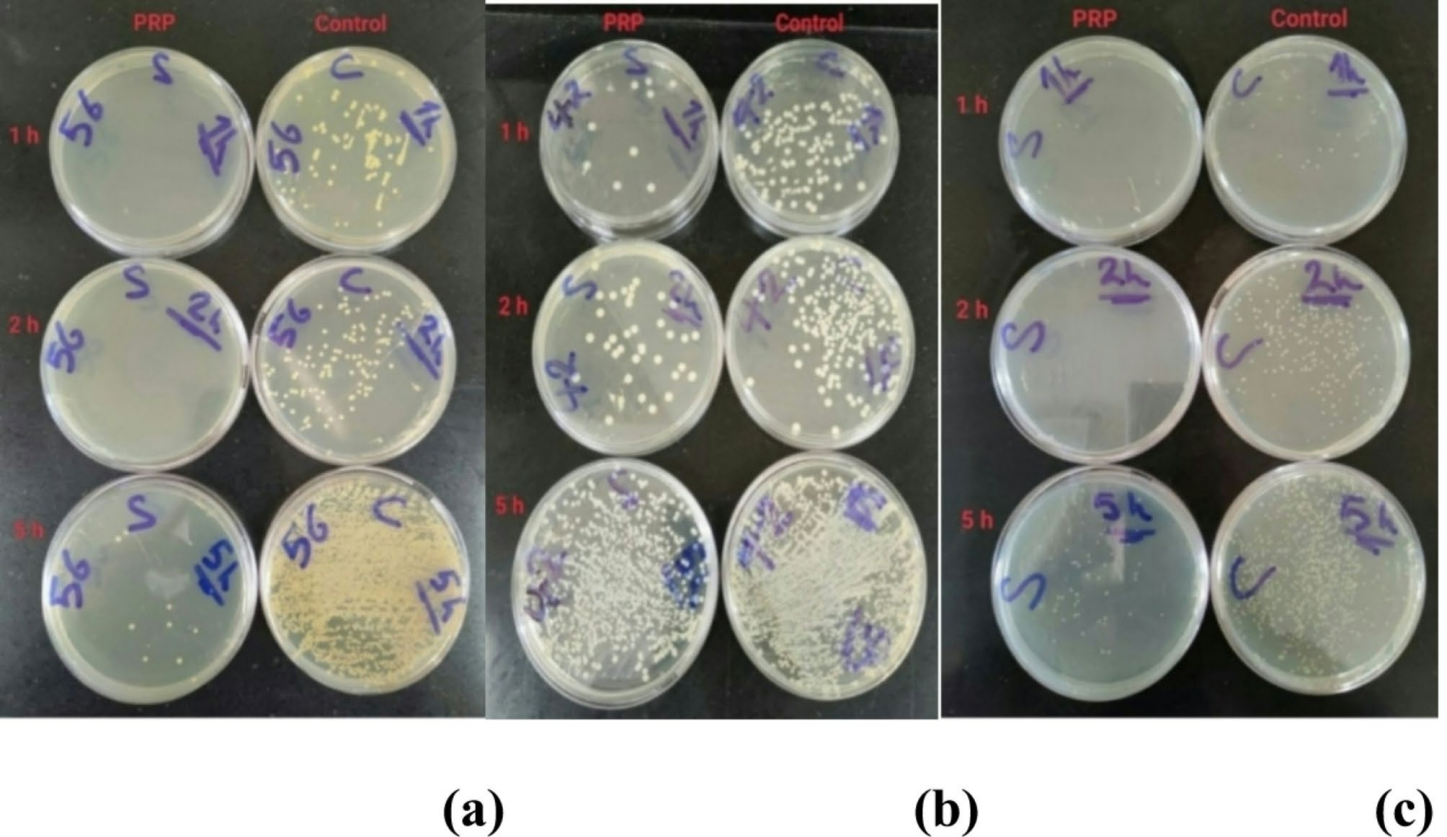
Colony counts of MRSA (**a**), MDR *K. pneumoniae* (**b**) and MDR *P. aeruginosa* (**c**) isolates from the PRP and control groups after 1, 2, and 5 h of incubation.

Colonies were enumerated using a colony counter following 24 h of incubation at 37 °C. Colony counts beyond 1000 were documented as 1000 CFU/mL (Fig. 13).

##### Determination of the antibiofilm activity of PRP

**Fig. 14:**
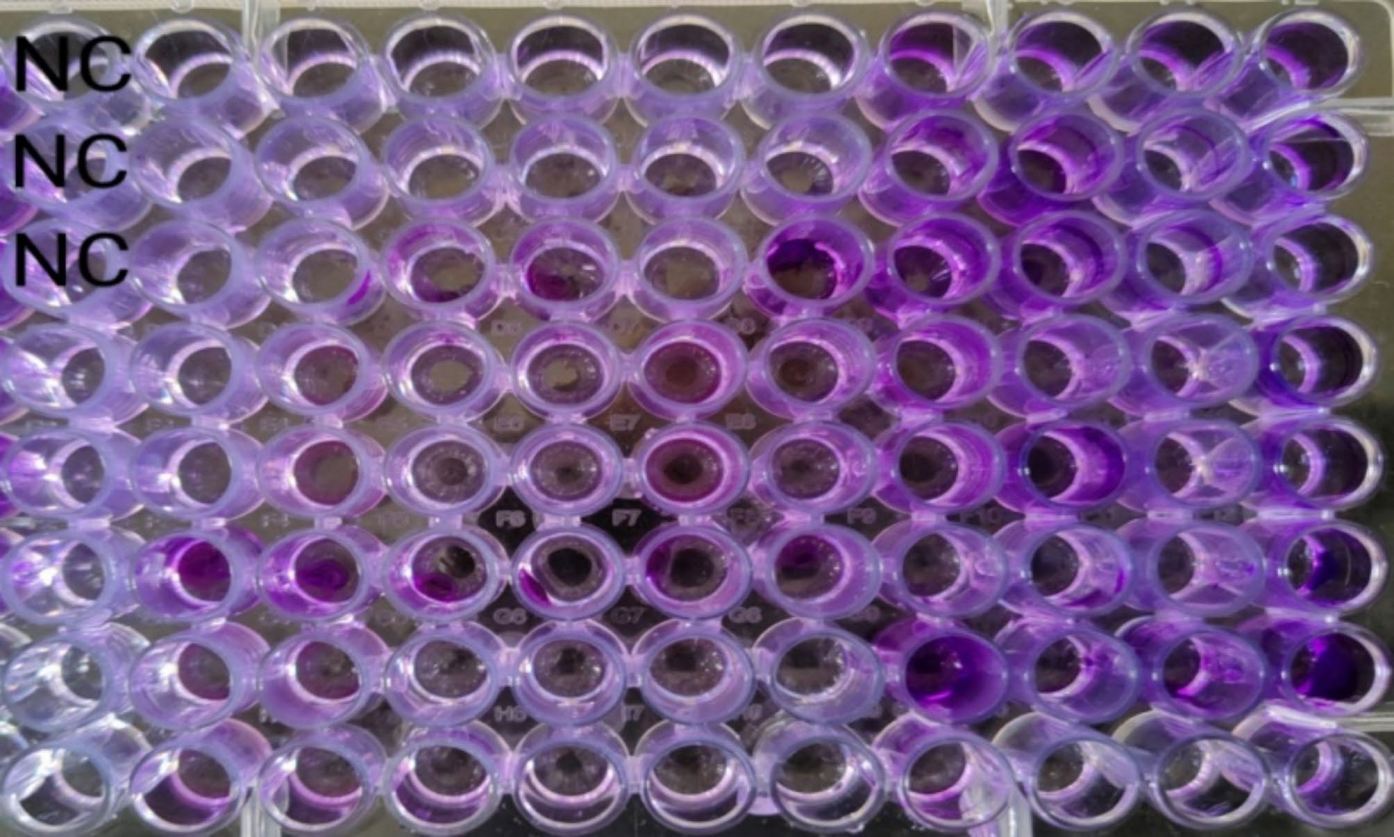
Crystal violet staining of bacterial biofilms: NC: negative control.

**Fig. 15:**
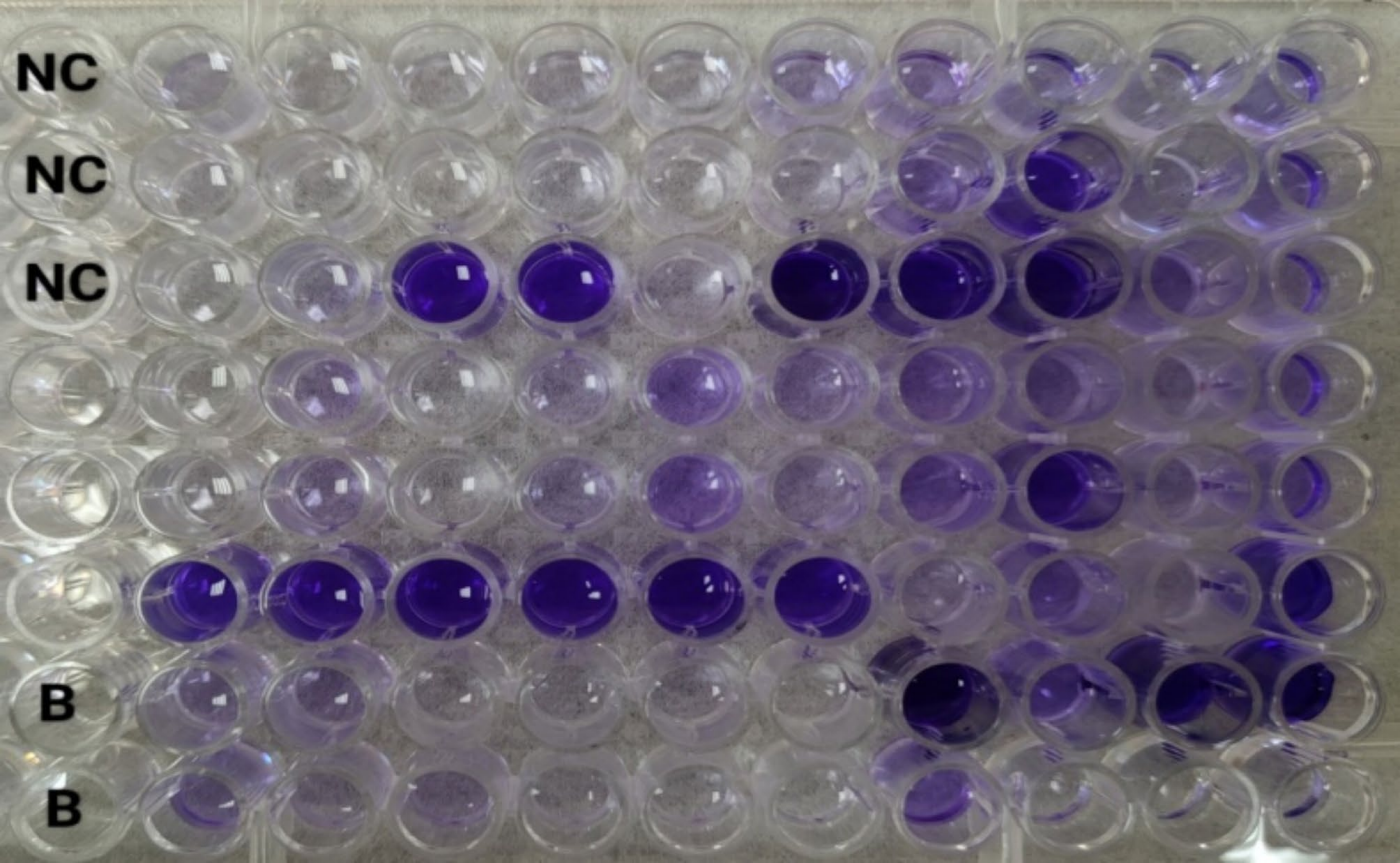
Crystal violet staining of bacterial biofilms after resolubilization with acetic acid and before the OD was measured: NC: negative control; B: blank.

*Detection of the biofilm-forming ability of the tested isolates*                                                               The biofilm-production ability of the identified isolates was evaluated using the tissue culture plate method, commonly referred to as the microtiter plate (MtP) assay (Figs. 14 and 15). The procedure involved culturing the samples in 96-well microtiter plates, followed by optical density (OD) measurement using a microplate enzyme-linked immunosorbent assay (ELISA) reader after staining the wells^71–73^. The cutoff OD (ODc) was determined by adding three standard deviations to the mean OD of the negative control. The bacterial isolates were classified according to the measured ODs as follows:

- OD ≤ ODc: non-biofilm producer (0).
- ODc ˂ OD ≤ 2×ODc: Weak biofilm producer (+).
- 2×ODc ˂ OD ≤ 4×OD: Moderate biofilm producer (++).
- 4×ODc ˂ OD: Strong biofilm producer (+++).

##### Effect of PRP on biofilm production (biofilm Inhibition assay)

**Fig. 16:**
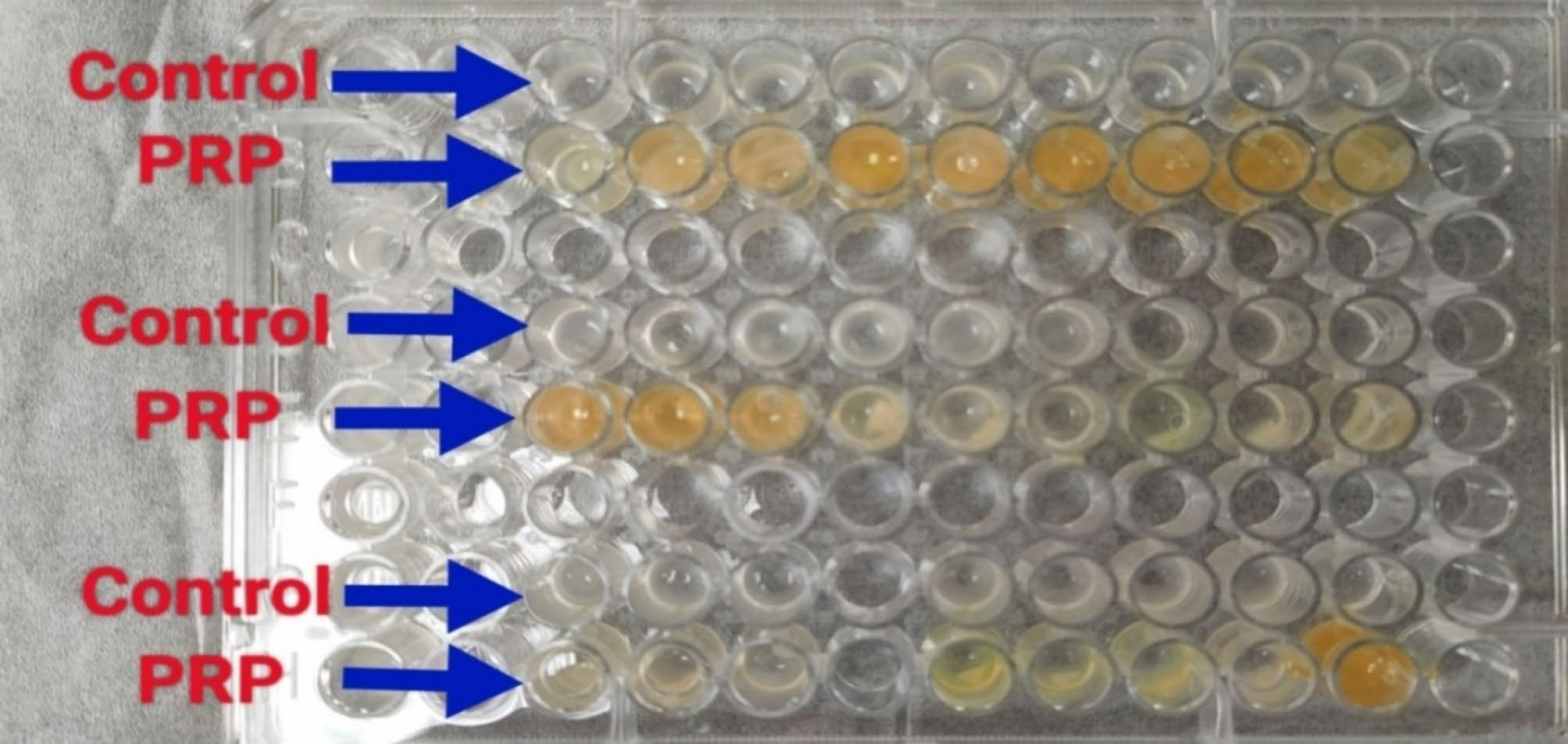
Biofilm inhibition assay after treatment with PRP: Control: inoculum without PRP; PRP: inoculum with PRP.

**Fig. 17:**
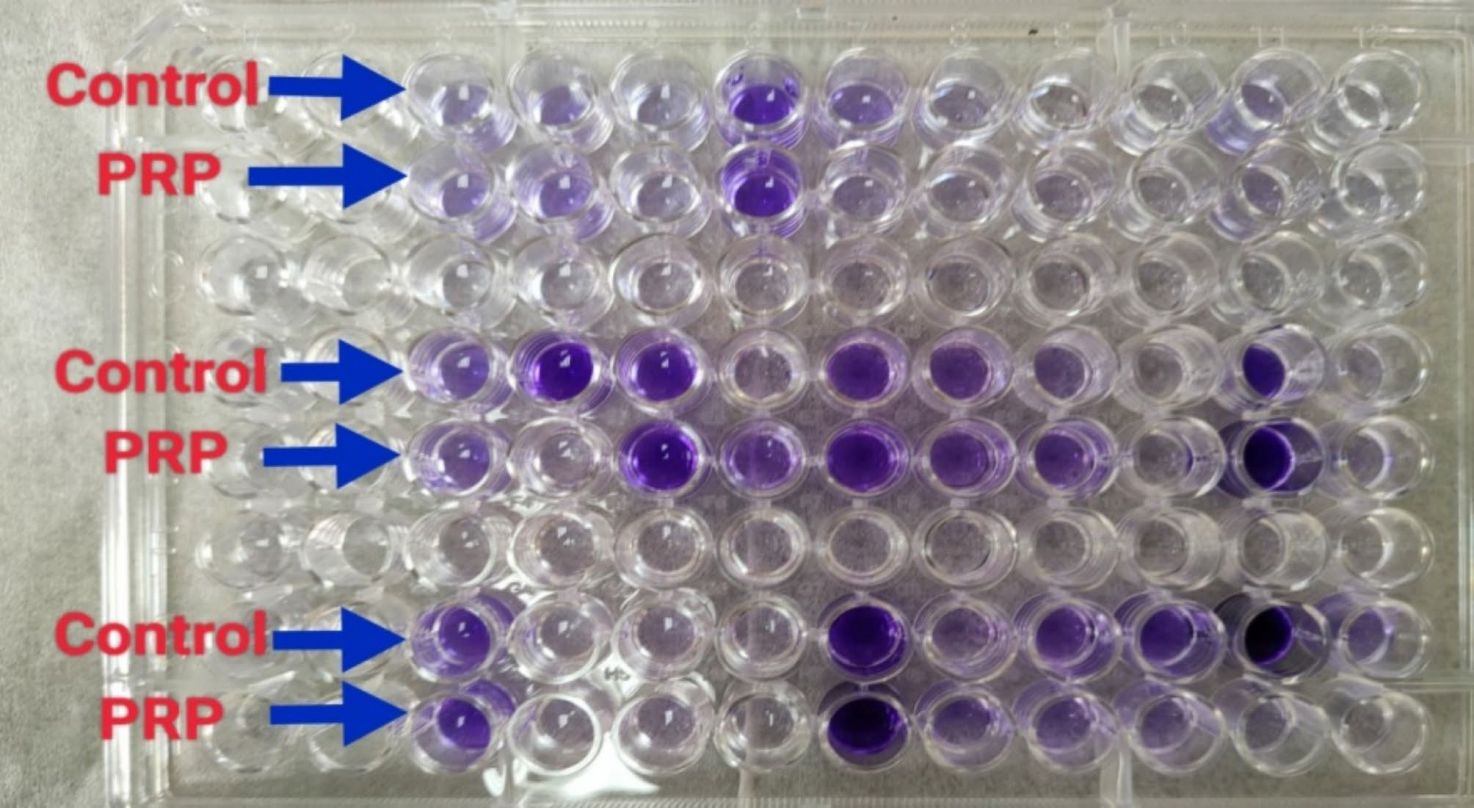
Crystal violet staining of biofilms after treatment with PRP: Control: inoculum without PRP; PRP: inoculum with PRP.

The inhibition of biofilm formation (by the biofilm-producing isolates) was assessed via the same procedure for detection of biofilm-production ability, except that 100 µL of activated PRP was added to 200 µL of diluted bacterial suspensions in each well (Figs. 16 and 17)^71,72^.

The absorbance values were measured with a microtiter plate reader at 630 nm. The extent of biofilm inhibition was determined in relation to the biofilm quantity developed without PRP (designated as 100% biofilm) and the media sterility control (designated as 0% biofilm).

It was observed that by inverting the plate to remove fluid from the wells, thick platelet gels remained at the bottom (leading to deep staining of most of the wells). Accordingly, abovementioned test was repeated but without staining, and assessment of viable biofilm cells by colony count was performed as follows^71^:

1. Two hundred microliters of tryptic soy broth (TSB) were added to the wells, and biofilm cells were suspended by vigorous pipetting.
2. The suspended biofilm was transferred to a new 96-well flat bottom microtiter plate.
3. One hundred microliters from each well were plated onto a TSA plate and incubated for 24 h.
4. The plates were then examined, and the colony count was recorded.
5. The number of colonies from the PRP wells were compared with those from the corresponding control wells (defined as 100% biofilm) and the negative control wells (defined as 0% biofilm).

##### Effect of PRP on mature biofilms (biofilm eradication assay)

**Fig. 18:**
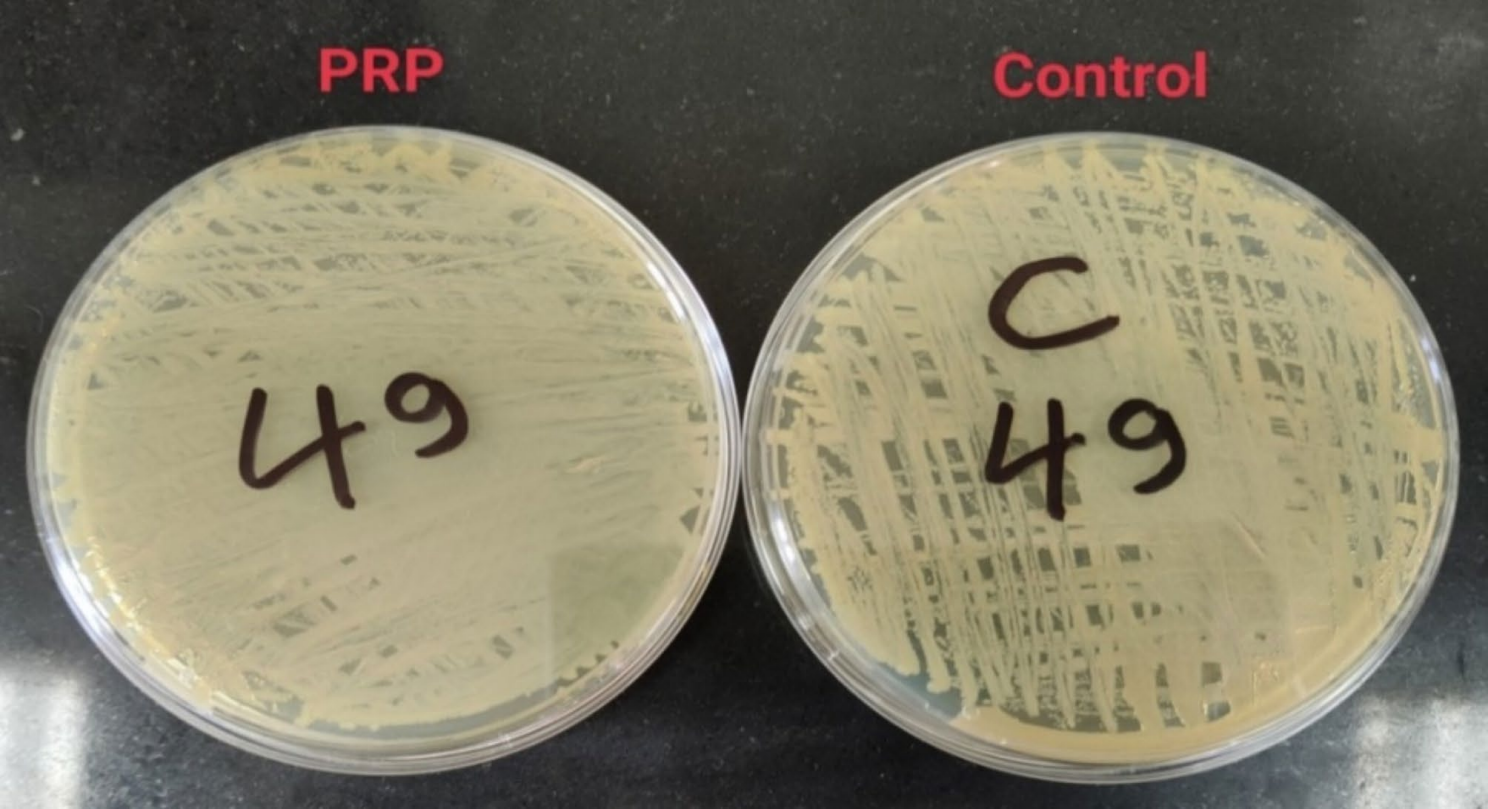
Colony counts in the biofilm eradication assay: PRP: biofilm treated with PRP; Control: biofilm not treated with PRP.

This test was conducted to assess the impact of PRP on existing biofilms, following the methodologies outlined by Cruz et al. and Haney et al.^71,72^. The assay was conducted as outlined below:

1. The biofilms were allowed to mature for 24 h as previously described.
2. The wells were aspirated and washed 3 times with distilled water.
3. One hundred microliters of activated PRP was added to each well, and the plate was incubated at 37 °C for 24 h.
4. The contents were then discarded, and the plates were washed with distilled water.
5. Approximately 200 µL of TSB was added each well, and the mixture was incubated for another 24 h.
6. After incubation, the biofilms were scraped from the wells via sterile tips, and 100 µL from each well was inoculated into a TSA plate and incubated for 24 h^73^.
7. The plates were then inspected, and the number of colonies was counted (Fig. 18).
8. Colony counts from the PRP wells were compared with those from the corresponding control wells and the negative control wells.
9. The test was repeated except that the plate was incubated after the addition of PRP for 2 h instead of 24 h.

### Statistical analysis

The data were analyzed using version 25.0 of the Statistical Package for Social Sciences (SPSS) software. Qualitative data were expressed in numerical form and percentages. The Kolmogorov-Smirnov test was employed to assess the normality of the distribution. The quantitative data were given as ranges (minimums and maximums), means, standard deviations, and medians. The significance of the acquired results was assessed at the 5% level.

## Data Availability

The datasets generated and/or analyzed during the current study are available from the corresponding author on reasonable request.
